# Design of a comprehensive protection algorithm for fault detection, classification, and location in high voltage direct current transmission lines connected to wind farms based on local terminal measurements

**DOI:** 10.1371/journal.pone.0356053

**Published:** 2026-09-24

**Authors:** Mahyar Abasi, Vahid Davatgaran, Morteza Azizi

**Affiliations:** 1 Department of Electrical Engineering, Faculty of Engineering, Arak University, Arak, Iran; 2 Research Institute of Renewable Energy, Arak University, Arak, Iran; 3 Department of Electrical Engineering, Technical and Vocational University (TVU), Tehran, Iran; 4 Department of Electrical Engineering, Faculty of Engineering, Arak University, Arak, Iran; 5 Operation Department, Electricity Distribution Company of Markazi Province, Arak, Iran; China University of Petroleum East China - Qingdao Campus, CHINA

## Abstract

With the increasing adoption of bipolar high-voltage direct-current (HVDC) transmission lines for wind farm integration, the development of fast, accurate, and communication-independent protection schemes has become a critical technical requirement. This paper proposes a comprehensive protection algorithm within an integrated single-ended framework that performs fault detection, fault classification, and fault location simultaneously using only local voltage and current measurements. In the detection stage, transient current signals are processed through common- and differential-mode decomposition to ensure fast and reliable fault inception identification. In the classification stage, the faulted pole and fault type are determined by exploiting the dynamic voltage patterns of the two poles. For fault location, an analytical model is developed based on the voltage–current relationships of the bipolar HVDC line, while explicitly accounting for self-resistance, mutual resistance, and inter-pole coupling effects. In addition, the protection thresholds are systematically defined using per-unit parameters and dimensionless indices, together with statistical criteria extracted from the system behavior under both normal and faulted conditions; this enhances their tunability, robustness, and applicability across different HVDC configurations. The proposed method is evaluated in the MATLAB/Simulink environment under varying operating conditions, fault resistance values, and line-parameter uncertainties. Simulation results show that the average and maximum fault detection delays are 3.32 ms and 4.96 ms, respectively. Moreover, the faulted pole is identified with complete accuracy, while the average and maximum fault-location errors are 0.69% and 0.98%, respectively, confirming the high speed, accuracy, and robustness of the proposed protection scheme.

## 1. Introduction

### 1.1 Research necessity and motivation

With the increasing deployment of HVDC technology for integrating renewable energy resources, particularly wind farms located far from major load centers, fast and reliable protection of DC transmission lines has become a critical challenge in modern power systems. The highly transient nature of faults in HVDC lines, together with the dynamic behavior of power electronic converters, makes fault detection, classification, and location in such systems more complex than in conventional AC networks, highlighting the importance of developing effective protection schemes [1]. Various approaches have been proposed in the literature for fault detection and location in HVDC transmission lines. A considerable portion of these methods is based on traveling wave analysis, where transient waves generated by a fault are utilized to determine the fault location along the transmission line [2]. Although traveling-wave-based methods can achieve high accuracy in fault location, they generally require high sampling rates, precise detection of wave arrival times, and in some cases coordination or synchronization between line terminals. In addition, several studies have proposed time-domain approaches based on mathematical modeling of the HVDC system to estimate the fault location. In these methods, the dynamic relationships of voltage and current signals are analyzed to determine the fault position along the transmission line [3]. While such techniques can provide satisfactory accuracy under certain operating conditions, their performance may be affected by variations in system parameters and network operating conditions. Furthermore, review studies indicate that advanced signal processing techniques and intelligent algorithms have also been investigated to enhance HVDC protection performance; however, many of these approaches show significant dependence on feature extraction procedures, training datasets, and specific network operating conditions [1]. This issue becomes even more critical in HVDC systems connected to renewable energy sources, particularly wind farms, where power fluctuations and dynamic variations can influence the reliability of protection schemes. Therefore, the development of a simple, fast, and accurate protection scheme that operates solely based on local measurements at a single terminal remains an important research need in this field. The main motivation of this study is to propose a comprehensive protection algorithm based on local terminal measurements, capable of rapidly detecting faults, identifying the fault type, and accurately locating the fault position in bipolar HVDC transmission lines, including PTG, NTG, and PTN faults, without relying on data exchange between line terminals or complex data-driven techniques.

### 1.2 Review of previous studies and research gaps

The research literature on fault detection, classification, and location in HVDC networks and hybrid AC–DC systems has expanded considerably in recent years. Considering the diversity of the proposed approaches, these studies can be systematically categorized into five main groups: feature extraction–based classical machine learning methods, deep learning–based methods, traveling-wave and high-frequency transient analysis methods, model-based and impedance-based methods, and advanced time–frequency transform–based methods. In the first category, feature extraction and classical machine learning methods [4–8], the primary focus is on extracting meaningful features from voltage and current signals and employing machine learning classifiers for fault detection and classification. In these approaches, signal processing tools are first used to extract features capable of distinguishing between healthy and faulty operating conditions, and these features are subsequently fed into classification algorithms. For instance, in [4], a heterogeneous framework based on a combination of SVM and KNN is proposed, where features such as local gradients, harmonic components, and correlation coefficients are extracted from single-ended signals and utilized for internal fault detection. Similarly, in [5], spectral analysis of voltage signals combined with artificial neural networks is employed to identify frequency patterns associated with faults in CSC-HVDC and VSC-HVDC systems. Furthermore, the study in [6] proposes an intelligent protection scheme for MT-HVDC networks in which time–frequency features extracted using the discrete wavelet transform and spectral energy based on Parseval’s theorem are fed into a neural network optimized using a Bayesian optimization algorithm, achieving high fault classification accuracy. In addition, [7] presents a method that combines FFT-based harmonic analysis, voltage feature extraction, and an OED–WOA–LSSVM classifier to detect single-phase-to-ground faults in hybrid AC–DC lines, demonstrating significant robustness against variations in fault location, transient resistance, and measurement noise. Moreover, in [8], the use of DOST and FDST transforms alongside PNN and BPNN networks enables the extraction of short-term transient features and significantly improves fault detection and location accuracy in six-pulse HVDC systems. Overall, these studies indicate that with appropriate feature extraction design and optimal classifier selection, classical feature-based methods can still provide fast, accurate, and reliable performance in HVDC and hybrid AC–DC environments. The second category includes deep learning–based methods [9–11], which leverage the ability of deep neural networks to automatically extract features from raw data, thereby reducing the limitations associated with manual feature engineering. Within this framework, deep networks can automatically identify complex structures and nonlinear relationships embedded in electrical signals. In [9], a method based on a convolutional neural network combined with the Fast Fourier Transform is proposed, where spectral features of voltage and current signals are first extracted and then fault-related patterns are identified through convolutional layers. Similarly, in [10], several neural network architectures are investigated for fault detection, classification, and location in a three-terminal VSC–HVDC system, and the results demonstrate that these methods can detect faults within a very short time and determine their location with acceptable accuracy. Furthermore, [11] proposes a hybrid CNN–SVM approach combined with feature extraction using the Hilbert–Huang transform. By integrating CNN’s capability for deep feature extraction with the accurate classification performance of SVM, this method achieves very fast and precise fault detection, classification, and location in multi-terminal VSC–HVDC systems. These studies indicate that deep learning can significantly enhance the accuracy and speed of data-driven protection systems. The third category includes traveling-wave and high-frequency transient analysis methods [12–16], which focus on exploiting high-frequency components generated at the moment of fault occurrence. When a fault occurs in HVDC lines, transient waves are generated and propagate along the transmission line at speeds close to the wave propagation velocity. Analyzing these waves enables very fast fault detection and location. In [12], a rapid fault detection and location method for HVDC lines is presented based on the arrival times of traveling waves at different terminals, with results showing that its accuracy is largely independent of fault resistance. In [13], wavelet transform is employed to extract high-frequency components of transient signals, which are then used for fault detection and classification in VSC-HVDC systems. The study in [14] introduces a two-terminal traveling-wave protection scheme capable of distinguishing between internal and external faults with very high speed by comparing transient information at both ends of the line. Additionally, [15] applies high-frequency transient analysis for fault detection in multi-terminal DC networks, demonstrating that features extracted from these components can rapidly identify the faulty region without requiring an accurate network model. Furthermore, [16] proposes an accurate fault location method based on high-sampling-rate sensors and traveling-wave processing, whose performance has been evaluated under noise, line parameter variations, and network topology changes, with results confirming its superiority in terms of speed and accuracy. Despite their significant advantages, the requirement for measurement equipment with very high sampling rates remains one of the main practical challenges of these methods. The fourth category includes model-based and impedance-based methods [17–20], in which fault detection and location are carried out based on physical system modeling and the analysis of voltage–current relationships. In these methods, the actual response of the system is compared with the expected behavior derived from analytical models, and fault-induced deviations are used for protection and location purposes. In [17], Mu et al. propose a fault location scheme for HVDC converters based on fault loop analysis, in which the fault loop is analytically characterized and the associated electrical quantities are employed to identify the fault location with high accuracy. Similarly, [18] presents an approach based on apparent line impedance and two-terminal voltage–current analysis for fault detection and location in HVDC transmission lines. Owing to their clear physical interpretation and relatively low measurement requirements, such methods remain widely used in HVDC protection schemes. Subsequently, [19] attempts to improve fault location accuracy under various operating conditions and in the presence of fault resistance by developing more detailed models of HVDC lines and converters. In addition to these classical approaches, more recent studies have focused on internal fault detection in MMC converters. In this regard, [20] presents a fault detection and location method for MMC converters in offshore wind power systems based on the analysis of circulating current harmonics and voltage differences among submodule capacitors, enabling accurate identification of internal submodule faults. Overall, model-based methods continue to play an important role in the development of protection and condition monitoring systems in HVDC networks due to their reliance on physical system principles and high interpretability. Finally, the fifth category consists of advanced time–frequency transform–based methods [21,22], which are developed to analyze non-stationary signals and extract transient features associated with fault events. In these approaches, tools such as wavelet transform and S-transform are used to simultaneously represent the temporal and frequency information of signals. In [21], the discrete wavelet transform is applied to decompose voltage and current signals into multiple frequency levels, and the resulting coefficients are used as indicators for fault detection and classification. Similarly, in [22], the S-transform is employed to obtain the time–frequency spectrum of measured signals, and the extracted features are used for fault detection and location in HVDC systems. Due to their strong capability in detecting fast transient phenomena and extracting accurate features from non-stationary signals, these methods are considered powerful tools in the design of protection and fault monitoring systems for HVDC and AC–DC networks.

The challenges and limitations highlighted in previous studies regarding fault detection and location in HVDC transmission lines can be summarized as follows:

- Dependence of existing methods on manual feature design and classifier selection, leading to reduced stability and generalization capability under real HVDC network conditions.
- Requirement of very high sampling rates for high-frequency transient and traveling-wave methods, posing significant limitations for practical implementation, particularly in lines connected to wind farms.
- Sensitivity of model-based and impedance-based approaches to operating condition variations, wind power fluctuations, and network parameter uncertainties, resulting in reduced accuracy and stability under real-world conditions.
- Lack of an integrated and interpretable framework based on single-ended measurements that simultaneously performs fault detection, classification, and location within a unified structure.

### 1.3 Contributions and innovations

This paper introduces a three-stage analytical framework for fault detection, classification, and location in HVDC transmission lines connected to wind farms. The framework is built upon a coherent mathematical formulation that has been fundamentally redesigned. The objective of this framework is to transfer the transient behavior of the system from the terminal level into a structured modeling space, enabling a physical–mathematical interpretation of transient phenomena using only single-ended data. In the first stage, the proposed method introduces two current quantities constructed from the combination and contrast of the positive and negative pole components, providing a modal reinterpretation of the dynamic behavior of the transmission line. In this representation, the current is analyzed not as raw data but through two independent mappings: one describing the common behavior of the two poles and the other representing their differential behavior. As a result, the three fundamental network states, healthy operation, single-pole faults, and pole-to-pole faults, naturally occupy three distinct dynamic regions. This structural separation allows fault detection to be performed based on the geometric behavior of the currents rather than relying on empirical threshold settings. The second stage focuses on the voltages of the two poles and exploits a key physical–mathematical principle: disturbances caused by faults alter the temporal pattern and magnitude of each pole voltage in a distinctive manner, and these variations act as identifiable signatures of the fault type. The formulation in this stage clarifies the relationship between the voltage response of the faulty pole and that of the healthy pole. Without requiring feature extraction or data-driven techniques, it distinguishes among the three fault categories, PTG, NTG, and PTN, based on predictable and coherent transient behaviors. This structure demonstrates that the modal analysis of the first stage naturally leads to a clear and reliable distinction in the voltage behavior analyzed in the second stage. In the third stage, fault location is performed based on a revised distributed-parameter model. In this model, voltage variations in the two poles are interpreted as carriers of information about the fault distance. The analysis shows that the difference between the extracted pole voltages exhibits a unidirectional and fully closed-form relationship with the fault location. This relationship emerges in a structured manner through the interaction between the line’s series and mutual resistances and the transient current components. The result is a single-ended fault locator that operates without requiring communication between line terminals and maintains inherent stability under severe transient conditions, high fault resistances, and fluctuations caused by wind generation. Together, these three formulations, modal current analysis, transient voltage interpretation for classification, and the direct mapping of voltage difference to fault location, form an integrated framework grounded in the physical behavior of the system. Rather than relying on complex signal processing techniques or communication-based schemes, the proposed approach is built upon a structural understanding of HVDC dynamics. The final outcome is an advanced and reliable single-ended protection method for HVDC networks connected to wind farms, capable of delivering stable and consistent performance under severe transient disturbances, source variations, and demanding operating conditions. The main innovations of the proposed method are summarized in the following highlights:

- Presentation of a unified framework based on an analytical formulation of a distributed-parameter model for fault detection, classification, and location using local measurements.
- Introduction of a modal redefinition of currents that forms stable and distinguishable dynamic regions for accurate fault identification.
- Development of an inter-pole transient voltage formulation that ensures reliable fault discrimination even under variable and irregular wind farm dynamics.
- Proposal of a single-ended fault location method based on the direct mapping of voltage difference to fault distance, which is robust against wind power fluctuations and network operational variations.

### 1.4 Organization of the paper

This paper is organized into five sections. Section 2 presents the theoretical foundation of the proposed method. In this section, the general structure of the studied network is first introduced, followed by the formulation of the three algorithms for fault detection, classification, and location. Finally, the proposed scheme is illustrated through a comprehensive flowchart. Section 3 presents the results obtained from software simulations of multiple fault scenarios. These results demonstrate the performance of the proposed algorithm through its software implementation. Section 5 provides a comparison between the proposed method and existing approaches reported in the literature. Finally, the concluding section presents the overall summary and final conclusions of the paper.

## 2. Proposed method

This section presents the overall structure of the proposed method for fault detection, classification, and location in a bipolar HVDC transmission line. First, the topology of the studied network and the measured signals used in the algorithm are introduced. Then, the analytical framework of the method is described, including fault detection based on current indicators, fault type identification using voltage variations, and finally fault distance estimation based on voltage–current relationships. Next, a comprehensive flowchart of the algorithm execution and its decision-making logic is presented. Finally, the procedure for determining the threshold values used in the algorithm is explained using a data-driven statistical approach.

### 2.1 General introduction to the studied network structure and research objectives

**Fig 1:**
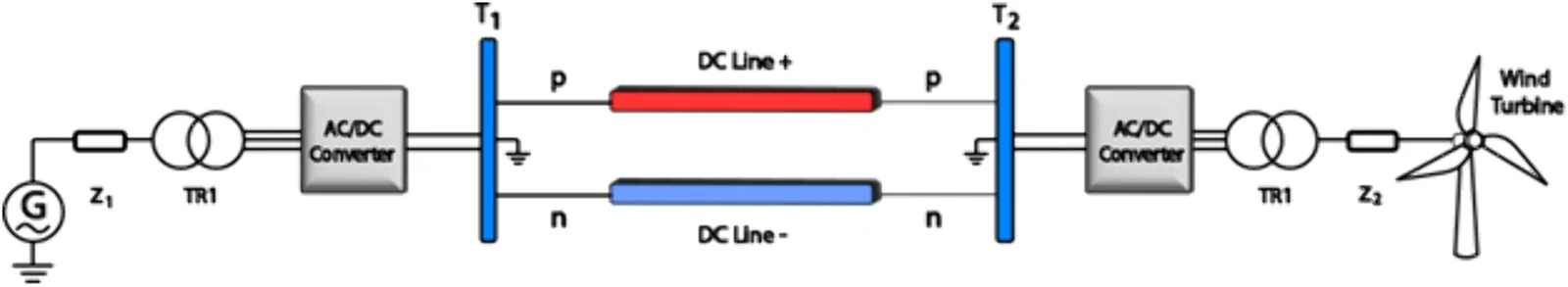
General topology diagram of the studied network.

The general structure of the network studied in this paper is a bipolar HVDC transmission system used to transfer power between an AC grid and a renewable energy–based generation source. As shown in Fig 1, this structure includes an AC network on the left side, which is connected to the first terminal of the HVDC system through an AC/DC converter. This terminal, denoted as T_1_, is responsible for converting the AC power of the grid into DC power and injecting it into the DC transmission link. After conversion, the electrical power is transmitted through a bipolar HVDC transmission line consisting of the positive pole (p) and the negative pole (n) toward the second terminal. On the opposite side, at terminal T_2_, a DC/AC converter is installed, which reconverts the transmitted power into AC power and delivers it to the wind farm. In addition to increasing the power transfer capacity, the use of a bipolar structure in HVDC lines provides more stable operation and greater flexibility for the system. Considering the long length of HVDC lines and the high sensitivity of these systems to faults, the design of a fast and accurate protection algorithm for fault detection, classification, and location is of particular importance. In this regard, the present paper proposes a protection method based on local measurements at terminal T_1_. In this method, the currents and voltages of the positive and negative poles at the T_1_ terminal bus are used for implementing the protection algorithm, including the currents $i_{T_{\text{1}}-p}$ and $i_{T_{\text{1}}-n}$, and the voltages $V_{T_{\text{1}}-p}$ and $V_{T_{\text{1}}-n}$. These quantities are used as the main input data of the algorithm, from which combined current and differential current indices are extracted as the basis for detecting the occurrence of faults in the system. After fault detection, the algorithm uses the voltage variations of the positive and negative poles to determine the type of fault that has occurred on the transmission line and classifies it into one of the PTG, NTG, and PTN categories. Subsequently, by utilizing current- and voltage-dependent relationships along with the electrical parameters of the line, the distance of the fault from the measuring terminal is calculated. The overall framework of this process is designed in the form of a hierarchical structure consisting of fault detection, fault classification, and fault location stages, and its operational logic will be presented through the comprehensive flowchart provided in this paper. This stepwise structure enables fast signal processing and accurate decision-making and, due to its reliance on local measurements, offers practical implementation capability in HVDC line protection systems.

### 2.2 Formulation of the proposed method

In this section, the analytical and computational framework of the proposed scheme for fault detection, classification, and location in a bipolar HVDC transmission system is presented. Considering that the transient behavior of current and voltage in HVDC lines is highly sensitive to pole-to-ground and pole-to-pole faults, it is essential to develop a multi-stage algorithm capable of rapidly and reliably identifying fault occurrences using only local measurements and operating independently of system operating conditions. Accordingly, the structure presented in this section consists of three main subunits, each performing a specific and complementary function. In the first stage (fault detection), the occurrence of a fault as well as its initiation time is determined by analyzing the combined behavior of the two pole currents measured at terminal T_1_ and by extracting differential and combined current indices. This stage is designed based on a data-driven threshold logic and is capable of distinguishing actual fault behavior from non-fault transient fluctuations. In the second stage (fault classification), the instantaneous voltages of the positive and negative busbars are used to identify the type of fault. Specifically, the three possible fault conditions, PTG, NTG, and PTN, are accurately determined based on voltage variations with respect to the threshold value Thr4. This stage plays a key role in selecting the appropriate equation used for fault location. In the third stage (fault location), the voltage difference between the two poles is calculated using voltage–current relationships derived from the distributed parameters of the transmission line. Based on the identified fault type, the distance of the fault from terminal T_1_ is then determined. This approach enables fast, single-ended fault location with strong robustness against severe transient conditions. Overall, these three stages operate within an integrated flowchart structure, forming an efficient protection tool capable of accurately detecting the occurrence of a fault, determining its type, and identifying its precise location along the transmission line. In the following subsections, each stage is described in detail.

#### 2.2.1 Fault detection algorithm.

In this subsection, the objective is to formulate the fault detection problem for the studied topology. To detect the occurrence of a fault and determine its initiation time, the current components measured at terminal T_1_ are utilized. For this purpose, two current indices, denoted as $i_{com}$ and $i_{diff}$, are defined. According to Eq. (1), these indices are calculated as follows:


$$\begin{array}{c} \left\{ \begin{array}{c} @li_{com}=\frac{|i_{T_{\text{1}}-p}+i_{T_{\text{1}}-n}|}{\text{2}}\\ i_{diff}=\frac{|i_{T_{\text{1}}-p}-i_{T_{\text{1}}-n}|}{\text{2}} \end{array}\right. \end{array}$$ (1)

**Fig 2:**
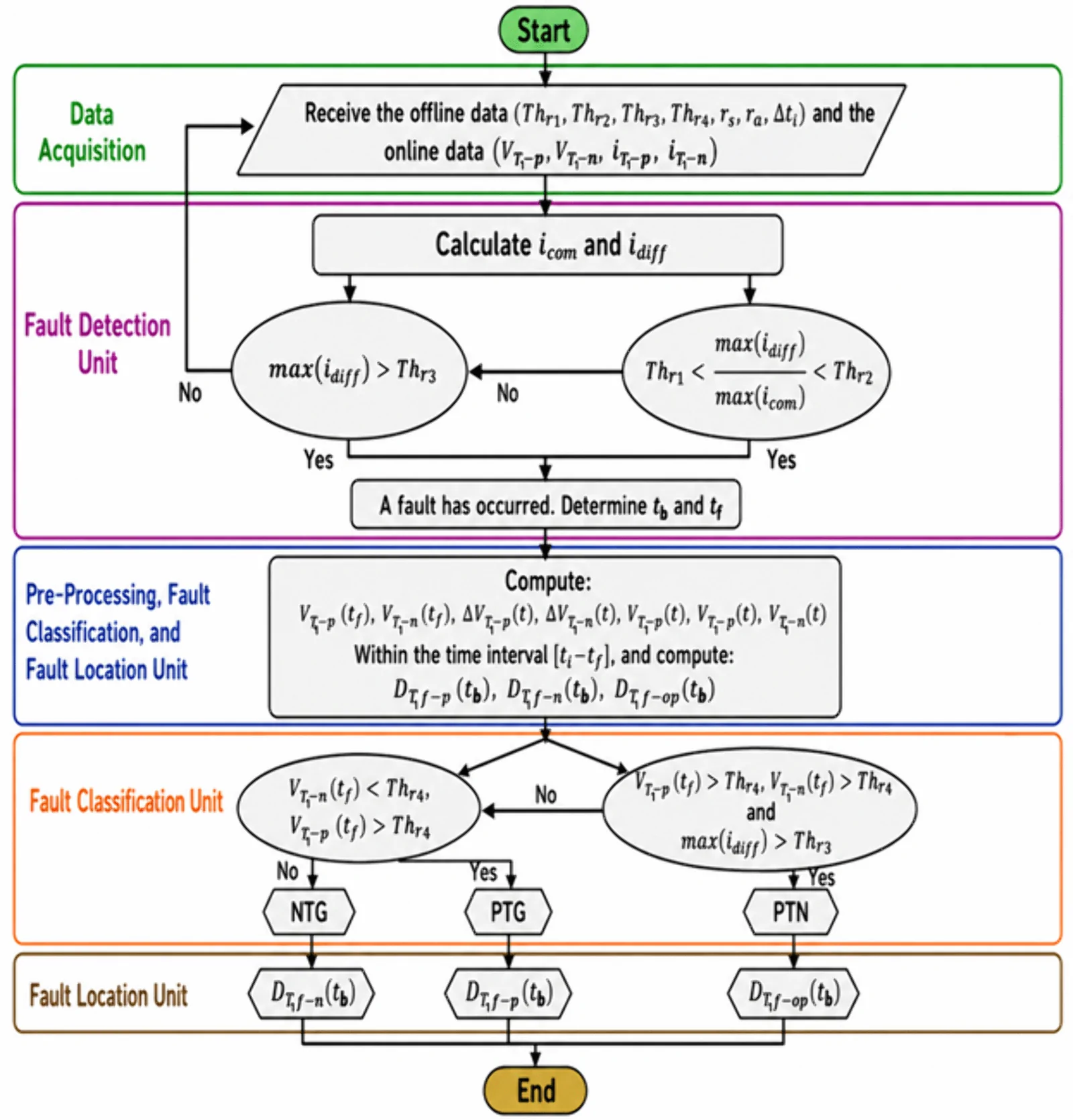
Comprehensive flowchart of the proposed scheme.

The parameter $i_{T_{\text{1}}-p}$ represents the current measured in the positive pole of the DC link at terminal T_1_, while $i_{T_{\text{1}}-n}$ represents the current measured in the negative pole of the DC link at the same terminal. The index $i_{com}$ denotes the common current component, obtained as half of the absolute value of the sum of the two pole currents. This index is used to reveal the common behavior of the two conductors, particularly under ground fault conditions. In contrast, the index $i_{diff}$ represents the differential current component, calculated as half of the absolute value of the difference between the pole currents, and reflects the degree of imbalance or behavioral difference between the positive and negative poles. The absolute value operator ∣·∣ ensures that the calculated indices are independent of current direction and are evaluated solely based on their magnitude. The factor $\frac{\text{1}}{\text{2}}$ is included to normalize the values resulting from the sum and difference of the two pole currents. If a PTG or NTG fault occurs, the ratio between $\text{max}(i_{com})$ and $\text{max}(i_{diff})$ will lie within a limited interval defined by the threshold values Thr_1_ and Thr_2_. In the case of a PTN fault, the value of $\text{max}(i_{diff})$ will exceed the threshold Thr3. Under normal operating conditions without faults, the value of $\text{max}(i_{com})$ remains approximately zero, and the value of $\text{max}(i_{diff})$remains below the threshold Thr_3_. To determine the threshold values, a data-driven threshold estimation technique is employed. Once a fault is detected, the fault initiation time $t_{f}$ is recorded as one of the inputs for the subsequent fault classification stage. The decision-making structure of this detection stage is illustrated through the logical conditions presented in the flowchart shown in Fig 2.

#### 2.2.2 Fault classification algorithm.

In this section, the identification of the faulted pole is carried out based on the analysis of the voltage behavior measured at terminal T_1_. From a physical perspective, the occurrence of a fault in the network disturbs the voltage balance of the system buses, which consequently produces noticeable variations in the voltage level of the corresponding poles. Therefore, monitoring the bus voltages can provide a direct indication of the location where the fault has occurred in the system. Considering the topology under study, three main types of faults may occur in the system, including PTN, PTG, and NTG. When a fault occurs in one of the poles, the current paths are altered, which leads to a significant increase or noticeable variation in the bus voltage of that pole compared to normal operating conditions. Accordingly, if the bus voltage of the considered pole exceeds the predefined threshold $Thr_{\text{4}}$, this voltage rise is interpreted as an indication of a fault occurring in that pole. Conversely, if the measured voltage does not exceed this threshold, it can be concluded that no fault has occurred in that pole. After identifying the pole involved in the fault, the next step is to determine the fault type. For this purpose, based on the operating regions defined in this study, a conditional decision-making logic is designed to distinguish the fault type among the PTN, PTG, and NTG cases. The structure of this decision-making process is presented step-by-step in the flowchart shown in Fig 2, which illustrates how the faults are classified according to the measured voltage values.

#### 2.2.3 Fault location algorithm.

In the proposed method, fault location is based on the direct relationship between the voltage drop occurring along the section between the measuring terminal and the fault point. After a fault occurs in the HVDC transmission line, the fault current inevitably flows through the electrical path between terminal T_1_ and the fault point. As these current passes through the line series impedance, a voltage drop is produced whose magnitude is proportional to the length of the path traveled to the fault location. Therefore, if the effective voltage drop per unit length is accurately determined, the relative fault distance can be inferred from the ratio of the terminal voltage measurement to the per-unit-length voltage drop. This principle constitutes the core of the proposed method and enables fault location estimation using only local measurements at terminal T_1_.

However, in bipolar HVDC lines, the voltage drop of each pole cannot be regarded as solely a function of the current of that same pole. In such a configuration, the positive and negative poles are not electrically completely independent, and due to the mutual effects arising from the physical arrangement of the conductors, current return paths, and coupling between the two poles, the current flowing in one pole can also influence the voltage drop observed on the other pole. Accordingly, in order to make the voltage-drop model more consistent with the actual line behavior, the effective voltage drop per unit length of each pole is considered as a combination of two components: the first component is associated with the current of the same pole and the line series resistance, and the second component is associated with the current of the opposite pole and the mutual coupling between the two poles.

Based on this, the effective voltage drop per unit length for the positive and negative poles at terminal T_1_ is defined, respectively, by Eqs. (2) and (3).


$$\begin{array}{c} \Delta V_{T_{\text{1}}-p}(t)={\text{i}}_{T_{\text{1}}-p}(t)r_{s}+{\text{i}}_{T_{\text{1}}-n}(t)r_{m} \end{array}$$ (2)

$$\begin{array}{c} \Delta V_{T_{\text{1}}-n}(t)=i_{T_{\text{1}}-n}(t)r_{s}+i_{T_{\text{1}}-p}(t)r_{m} \end{array}$$ (3)

In the above equations, $i_{T_{\text{1}}-p}(t)$ and $i_{T_{\text{1}}-n}(t)$ denote the measured currents of the positive and negative poles at terminal T_1_, respectively. Furthermore, $r_{s}$ represents the per-unit-length series resistance of each pole, while $r_{m}$ denotes the resistive component associated with the mutual effect between the two poles. In Eq. (2), the term $i_{T_{\text{1}}-p}(t)r_{s}$ represents the direct contribution of the positive-pole current to the voltage drop of the same pole, whereas the term $i_{T_{\text{1}}-n}(t)r_{m}$ models the influence of the negative-pole current on the voltage drop of the positive pole. Similarly, in Eq. (3), the term $i_{T_{\text{1}}-n}(t)r_{s}$ represents the direct effect of the negative-pole current, while the term $i_{T_{\text{1}}-p}(t)r_{m}$ accounts for the mutual effect of the positive-pole current. Therefore, Eqs. (2) and (3) are not merely auxiliary expressions for the numerical calculation of the voltage drop; rather, they constitute the physical basis of the fault-location algorithm. If the mutual effect represented by $r_{m}$is neglected, the model reduces to a simplified form in which each pole is assumed to be independent of the other. Such an assumption in a bipolar HVDC line may introduce systematic errors in distance estimation, since part of the voltage drop observed in each pole originates from the current flowing in the opposite pole. Hence, the inclusion of the mutual terms in Eqs. (2) and (3) allows the calculated voltage drop to more closely represent the actual voltage distribution along the line [23].

Now, assume that the fault occurs at a relative distance $D_{T_{\text{1}}f}$ from terminal T_1_. In this case, if the effective per-unit-length voltage drop in the positive pole is $\Delta V_{T_{\text{1}}-p}(t)$, the cumulative voltage drop from terminal T_1_ to the fault location in the positive pole is given by $\Delta V_{T_{\text{1}}-p}(t)D_{T_{\text{1}}f}$. Similarly, the cumulative voltage drop in the negative pole is $\Delta V_{T_{\text{1}}-n}(t)D_{T_{\text{1}}f}$. Therefore, the voltage measured at the terminal can be expressed as the sum of the voltage drop along the path from the terminal to the fault location and the residual voltage at the fault point. Specifically, for the positive and negative poles, this relationship is formulated in Eq. (4) and Eq. (5), respectively:


$$\begin{array}{c} V_{T_{\text{1}}-p}(t)=\Delta V_{T_{\text{1}}-p}(t)D_{T_{\text{1}}f}+V_{f-p}(t) \end{array}$$ (4)

$$\begin{array}{c} V_{T_{\text{1}}-n}(t)=\Delta V_{T_{\text{1}}-n}(t)D_{T_{\text{1}}f}+V_{f-n}(t) \end{array}$$ (5)

In the above equations, $V_{f-p}(t)$ and $V_{f-n}(t)$ represent the voltages at the fault point from the perspectives of the positive and negative poles, respectively. These two terms indicate that, in a realistic scenario, the terminal voltage is not solely a result of the line voltage drop, but is also influenced by the residual voltage at the fault point. In metallic faults or low-resistance faults, the voltage at the fault point on the faulted pole is typically very small; however, in high-resistance faults, this voltage is non-zero and can affect the accuracy of the distance estimation. This point will be addressed later in the analysis of the fault resistance effect.

For a PTG fault, the primary fault current path is established from the positive pole to the ground. In this case, the positive pole is the faulted pole, and its voltage at the fault point tends toward a small value due to the ground. Therefore, in the initial derivation, it can be assumed that Eq. (6) holds:


$$\begin{array}{c} V_{f-p}(t)\approx \text{0} \end{array}$$ (6)

Consequently, the voltage equation of the positive pole at terminal T_1_ is simplified as Eq. (7):


$$\begin{array}{c} V_{T_{\text{1}}-p}(t)\approx \Delta V_{T_{\text{1}}-p}(t)D_{T_{\text{1}}f} \end{array}$$ (7)

By dividing both sides of Eq. (7) by $\Delta V_{T_{\text{1}}-p}(t)$, the fault distance from the perspective of the positive pole is obtained according to Eq. (8):


$$\begin{array}{c} D_{T_{\text{1}}f}=\frac{V_{T_{\text{1}}-p}(t)}{\Delta V_{T_{\text{1}}-p}(t)} \end{array}$$ (8)

Eq. (8) indicates that, in a PTG fault, the measured voltage of the positive pole at terminal T_1_ mainly represents the voltage drop developed along the path between the terminal and the fault location. Therefore, the ratio of this voltage to the effective per-unit-length voltage drop of the same pole determines the relative fault distance. This relation is not merely an empirical choice; rather, it follows from the physical fact that, in a PTG fault, the positive pole carries the dominant fault current and its voltage is directly affected by the fault location.

In contrast, in a PTG fault, the negative pole is generally considered the healthy pole, and the corresponding voltage at the fault point does not necessarily tend to zero. If the negative-pole equation is used for this type of fault, the considerable residual voltage on the healthy pole enters the calculation and may cause the estimated distance to deviate from the actual value. To clarify this issue, if the negative pole is used, the general relation can be written as Eq. (9):


$$\begin{array}{c} \frac{V_{T_{\text{1}}-n}(t)}{\Delta V_{T_{\text{1}}-n}(t)}=D_{T_{\text{1}}f}+\frac{V_{f-n}(t)}{\Delta V_{T_{\text{1}}-n}(t)} \end{array}$$ (9)

Since $V_{f-n}(t)$ on the healthy pole is usually not negligible, the second term can increase the estimation error. For this reason, in a PTG fault, the relation based on the positive pole, i.e., Eq. (8), is a more appropriate choice for fault location.

Similarly, in an NTG fault, the dominant fault current path is established through the negative pole. Under this condition, the negative pole is the faulted pole, and the voltage at the fault point on this pole decreases to a very small value. Therefore, the approximation in Eq. (10) can be used:


$$\begin{array}{c} V_{f-n}(t)\approx \text{0} \end{array}$$ (10)

As a result, the voltage equation of the negative pole at terminal T_1_ is simplified as Eq. (11):


$$\begin{array}{c} V_{T_{\text{1}}-n}(t)\approx \Delta V_{T_{\text{1}}-n}(t)D_{T_{\text{1}}f} \end{array}$$ (11)

By dividing both sides of Eq. (11) by $\Delta V_{T_{\text{1}}-n}(t)$, the relative fault distance for an NTG fault is calculated according to Eq. (12):


$$\begin{array}{c} D_{T_{\text{1}}f}=\frac{V_{T_{\text{1}}-n}(t)}{\Delta V_{T_{\text{1}}-n}(t)} \end{array}$$ (12)

Eq. (12) expresses the same physical rationale as Eq. (8), but for the negative pole. In this case, $V_{T_{\text{1}}-n}(t)$ reflects the voltage drop along the path between terminal T_1_ and the fault location in the negative pole. Therefore, the ratio of this voltage to the per-unit-length voltage drop of the same pole determines the fault distance. As in the previous case, using the non-faulted pole may lead to estimation deviation due to the presence of residual voltage at the fault point. Hence, in single-pole-to-ground faults, selecting the faulted pole for distance calculation is an essential requirement for preserving the accuracy of the algorithm.

In a PTN fault, the situation is different, since both poles are simultaneously involved in the fault-current path. In this type of fault, neither the positive pole nor the negative pole alone contains all the information required for fault location, because both poles contribute to the formation of the fault path, and each pole can provide an independent estimate of the fault distance. The two initial estimates are obtained from Eqs. (13) and (14):


$$\begin{array}{c} D_{p}(t)=\frac{V_{T_{\text{1}}-p}(t)}{\Delta V_{T_{\text{1}}-p}(t)} \end{array}$$ (13)

$$\begin{array}{c} D_{n}(t)=\frac{V_{T_{\text{1}}-n}(t)}{\Delta V_{T_{\text{1}}-n}(t)} \end{array}$$ (14)

In the ideal case, if the parameters of the two poles are perfectly symmetrical, the measurements are error-free, and the fault resistance does not introduce any asymmetric effect, the two estimates obtained from Eqs. (13) and (14) are expected to be identical. However, under practical conditions, factors such as slight asymmetry in line parameters, differences between the pole currents, coupling effects, measurement noise, and fault resistance may cause the two estimates, $D_{p}(t)$ and $D_{n}(t)$, to differ from each other. In such a situation, relying on the estimate of only one pole may make the fault-location result sensitive to disturbances or errors associated with that pole.

To reduce this sensitivity, in a PTN fault, the fault distance is calculated as the average of the two estimates obtained from the positive and negative poles, as given in Eq. (15):


$$\begin{array}{c} D_{T_{\text{1}}f}=\frac{\text{1}}{\text{2}}\left(\frac{V_{T_{\text{1}}-p}(t)}{\Delta V_{T_{\text{1}}-p}(t)}+\frac{V_{T_{\text{1}}-n}(t)}{\Delta V_{T_{\text{1}}-n}(t)}\right) \end{array}$$ (15)

Eq. (15) follows from the fact that, in a PTN fault, information related to the fault location is distributed across both poles. Therefore, the simultaneous use of both poles can partially mitigate the effects of local errors, parameter asymmetry, and measurement noise. In other words, the averaging operation in Eq. (15) is not merely a simple mathematical manipulation; rather, it is a strategy for combining two independent observations of a common physical phenomenon. This structure allows the estimate from one pole to compensate, to some extent, for any overestimation or underestimation that may occur in the estimate from the other pole due to slight asymmetric effects.

By substituting Eqs. (2) and (3) into the fault-location equations, the explicit form of the distance estimation can be obtained.

• For a PTG fault, Eq. (8) can be rewritten as Eq. (16):


$$\begin{array}{c} D_{T_{\text{1}}f}=\frac{V_{T_{\text{1}}-p}(t)}{i_{T_{\text{1}}-p}(t)r_{s}+{\text{i}}_{T_{\text{1}}-n}(t)r_{m}} \end{array}$$ (16)

• For an NTG fault, the corresponding form is obtained as Eq. (17):


$$\begin{array}{c} D_{T_{\text{1}}f}=\frac{V_{T_{\text{1}}-n}(t)}{i_{T_{\text{1}}-n}(t)r_{s}+i_{T_{\text{1}}-p}(t)r_{m}} \end{array}$$ (17)

• For a PTN fault, the expanded form of Eq. (15) is given by Eq. (18):


$$\begin{array}{c} D_{T_{\text{1}}f}=\frac{\text{1}}{\text{2}}[\frac{V_{T_{\text{1}}-p}(t)}{i_{T_{\text{1}}-p}(t)r_{s}+{\text{i}}_{T_{\text{1}}-n}(t)r_{m}}+\frac{V_{T_{\text{1}}-n}(t)}{{\text{i}}_{T_{\text{1}}-n}(t)r_{s}+{\text{i}}_{T_{\text{1}}-p}(t)r_{m}}] \end{array}$$ (18)

These explicit forms show that, in the proposed method, fault location is directly dependent on the voltages and currents measured at terminal T_1_ and does not require simultaneous measurements from the remote terminal. The presence of $r_{m}$ in the denominators of the equations also indicates that the coupling effect between the two poles is incorporated throughout all stages of the distance calculation.

The validity of the presented equations relies on several main assumptions. First, it is assumed that the line parameters remain constant or undergo negligible variations during the studied time interval. In other words, $r_{s}$ and $r_{m}$ are assumed to have well-defined values along the line and over the analysis window, making them suitable for voltage-drop calculation. Second, the fault-location equations are derived based on the dominant voltage and current components after the occurrence of the fault. Therefore, during the very initial instants following the fault, when fast transients, travelling waves, line capacitive effects, and converter responses may be significant, the simple ratio of voltage to per-unit-length voltage drop may be associated with a larger error. Third, in the initial derivation of Eqs. (8) and (12), it is assumed that the voltage at the fault point on the faulted pole is small compared with the voltage drop along the path. This assumption is more valid for low-resistance faults, whereas in resistive faults, the effects of $V_{f-p}(t)$ and $V_{f-n}(t)$ should be considered in the fault analysis.

Nevertheless, the general structure of Eqs. (8)–(15) remains physically valid, because these equations are directly derived from the voltage balance between terminal T_1_ and the fault location. In fact, the per-unit-length voltage drop of each pole is first calculated by considering both the direct effect of the current flowing in the same pole and the mutual effect of the current flowing in the opposite pole. Then, depending on the fault type, the pole or poles involved in the fault-current path are selected: the positive pole for a PTG fault, the negative pole for an NTG fault, and, for a PTN fault, the average of the estimates from both poles is adopted as the final fault location due to the simultaneous participation of both poles.

Consequently, Eqs. (8)–(15) provide a fault-location framework based on the physical model of voltage drop. While maintaining computational simplicity, this framework incorporates the coupling effect between the poles through Eqs. (2) and (3), and enables fault-location estimation using only local data measured at terminal T_1_. Therefore, the proposed formulation is not only computationally simple and implementable, but also physically interpretable, establishing a clear relationship among the measured voltage, the per-unit-length voltage drop, and the fault distance.

### 2.3 Comprehensive flowchart of the proposed scheme

In this section, the overall structure of the proposed method for fault detection, classification, and location in a bipolar HVDC transmission line is presented. First, the topology of the studied network and the measurement signals used in the algorithm are introduced. Then, the analytical framework of the method is described, including the stages of fault detection based on current-based indices, fault type identification using voltage variations, and finally fault distance estimation based on voltage–current relationships. Subsequently, the comprehensive flowchart of the algorithm execution and its decision-making logic is presented. Finally, the process of determining the threshold values used in the algorithm is explained using a data-driven statistical approach.

#### 2.3.1 Structuring of the proposed method.

The flowchart in Fig 2 presents a fully structured and hierarchical framework for monitoring, detecting, and locating faults in power systems. By integrating offline and online data, it establishes an intelligent decision-making mechanism that is highly sensitive to transient variations in the network. The process begins with receiving a set of reference data, including calibrated thresholds and characteristic coefficients. These thresholds are predetermined based on system modeling, operating conditions, and analysis of the network’s dynamic behavior, and they play a crucial role in preventing false fault detection caused by load fluctuations or non-fault disturbances. Subsequently, the real-time system data, including the currents and voltages measured at the two terminals, are acquired, and the combined current $i_{com}$ is calculated as a sensitive diagnostic index. This index, obtained through a modal or differential combination, enhances the visibility of variations caused by the occurrence of a fault. The fault detection unit employs a multi-layer threshold logic in which the maximum magnitude of the differential current is first compared with the threshold $Thr_{\text{3}}$, and then its ratio to the combined current is evaluated within a predefined range between $Thr_{\text{1}}$ and $Thr_{\text{2}}$. This two-level mechanism improves the capability of distinguishing transient disturbances from actual faults and reduces false positives. After the occurrence of a fault is confirmed, the fault initiation and termination instants are extracted, and the system proceeds to the preprocessing stage where the voltage differences are calculated within the critical time interval $\left[t_{f}-t_{b}\right]$. Subsequently, the terminal voltages and phase voltage differences, together with distance-related indices such as $D_{T_{\text{1}}f}(t_{b})$ and $D_{T_{\text{1}},np}(t_{b})$, are calculated. These indices represent a chain of current–voltage relationships that exhibit high sensitivity to the spatial location of the fault and are selected according to the fault type. The fault classification unit then distinguishes among the three categories NTG, PTG, and PTN using the threshold $Thr_{\text{4}}$ and by analyzing the voltage conditions at the final instant. This approach is based on analyzing voltage drops and phase symmetry or asymmetry, and when combined with the behavior of the differential current, it enables effective discrimination among phase-to-ground faults, phase-phase faults, and more severe fault conditions. Finally, the fault location unit determines the distance of the fault from the terminal by selecting the appropriate index according to the fault type. The obtained value is then converted into a percentage of the line length or a line coordinate, thereby providing useful information for protection and system operation. The integration of the different stages in this flowchart, the use of multi-stage thresholds derived from prior analysis, the reliance on sensitive current–voltage indices, and the implementation of a discriminative decision-making logic transform the algorithm into a precise and disturbance-resilient structure suitable for real-time implementation in intelligent protection systems.

#### 2.3.2 Threshold determination method.

**Fig 3:**
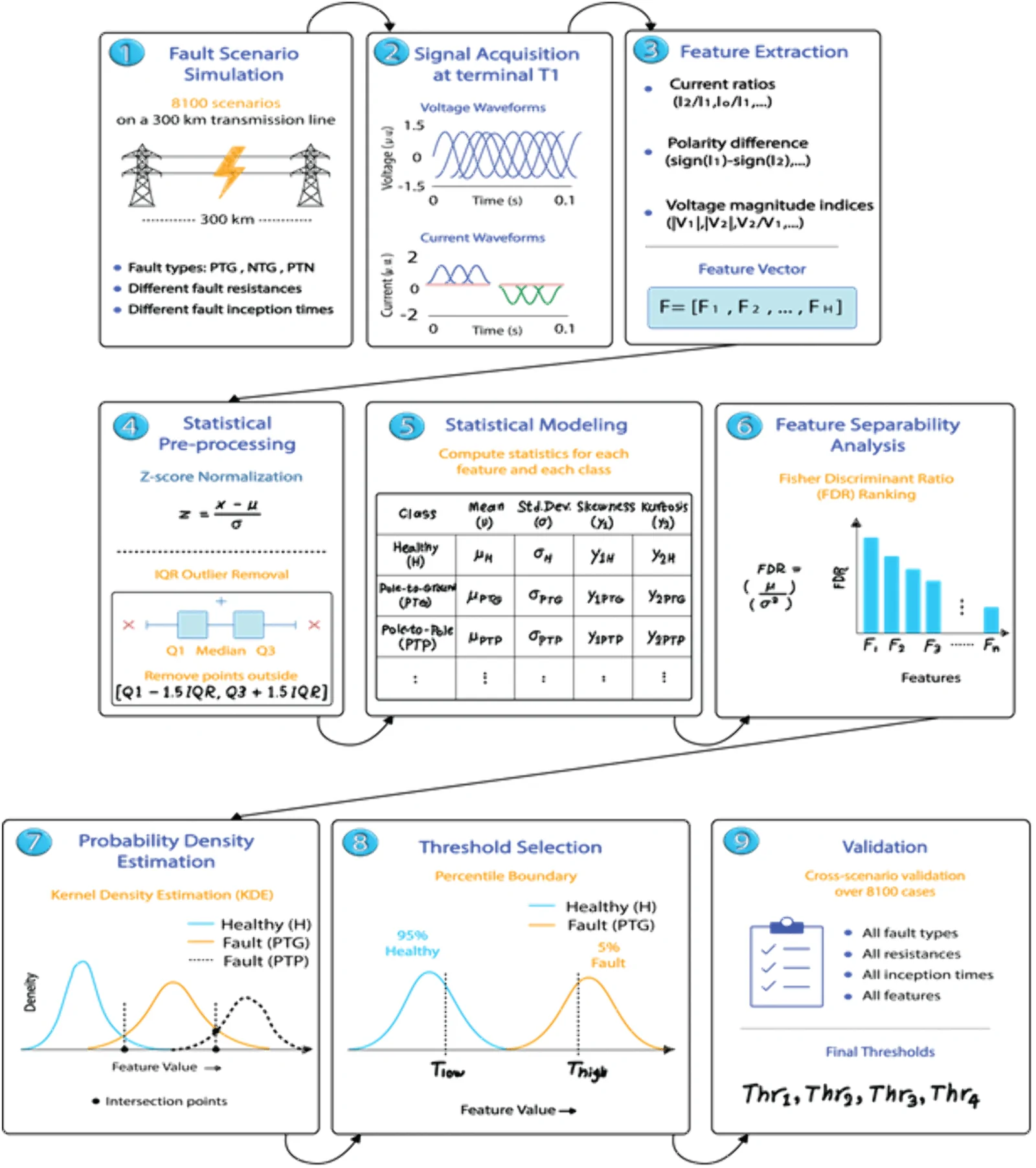
Overall process of the proposed statistical method for determining the threshold values $Thr_{1}$, $Thr_{2}$, $Thr_{3}$, and $Thr_{4}$ based on the analysis of data obtained from simulated fault scenarios and the distribution of extracted features.

In this study, the threshold values are determined based on a data-driven statistical threshold-selection framework implemented through several consecutive stages, allowing the decision boundaries to be systematically extracted from simulation data. The overall process is illustrated in Fig 3 and is described as follows. To this end, a total of 8,100 fault scenarios were first simulated for a 300 km transmission line, including PTG, NTG, and PTN faults occurring at different locations along the line. For each fault type, three fault resistance values (1, 50, and 100 Ω) and three different fault inception times were considered. The output of each simulation consisted of the current and voltage signals measured at terminal T_1_, which were considered as the raw dataset. In the first step, a set of diagnostic indices $F_{i}$ was extracted from these signals. These indices include current ratios, pole differential current indices, and voltage magnitude indices. They are computed using the RMS values, peak magnitudes, or amplitude variations of the signals within the time interval following fault occurrence.

Ultimately, for each simulation scenario, a multidimensional feature vector containing the collection of these indices was formed, providing the basis for the subsequent statistical analyses. During the statistical preprocessing stage, the feature scales were first unified using the Z-score normalization method. In this technique, each feature value is scaled relative to its mean and standard deviation across the entire dataset so that all features follow a distribution with zero mean and unit standard deviation. This normalization prevents features with larger numerical ranges from dominating the statistical analysis process. Next, an outlier detection process based on the IQR was applied to remove abnormal data points. In this method, the first quartile $Q_{\text{1}}$ and the third quartile $Q_{\text{3}}$ of the data are computed, and the IQR is defined as a measure of the central dispersion of the data. Data samples that fall outside the statistical interval defined by this range are identified as outliers and removed. This step reduces the influence of unrealistic values caused by transient oscillations or simulation artifacts on the statistical analysis.

After data refinement, the statistical modeling stage was carried out. The data were categorized into three system conditions: healthy state, pole-to-ground faults, and PTN faults. For each diagnostic index, the statistical distribution of the data was analyzed by calculating parameters such as the mean, standard deviation, skewness, and kurtosis in order to characterize the distribution shape, dispersion level, and asymmetry of the data. This statistical analysis provides a quantitative description of the behavior of each index under different system conditions. In the next step, the FDR was used to evaluate the capability of each index in separating different classes. This statistical metric is a common method in feature separability analysis and conceptually measures the ratio between the distance of the mean values of an index in two different classes and the dispersion of that index within those classes. Therefore, a larger FDR value indicates a stronger ability of that index to statistically distinguish between different system states. Based on this criterion, the indices with the highest FDR values were selected as the most effective features for the threshold determination process.

In the decision boundary determination stage, the probability distribution of each index for different classes was first estimated using the KDE method. KDE is a non-parametric technique for estimating the probability density function without assuming a predefined distribution shape. Instead, it approximates the actual data distribution using kernel functions, typically Gaussian kernels. After estimating the probability densities, the intersection points between the probability distributions of different classes were identified. Statistically, these intersection points correspond to locations where the probability of belonging to two classes is approximately equal; therefore, they were considered as initial candidates for the threshold values. In the subsequent step, in order to improve the stability and generalization capability of the thresholds, their exact positions were adjusted using a percentile-based boundary selection method. In this approach, statistical percentiles are used to define the decision boundary such that the thresholds are placed within the interval between the upper percentile of the healthy state distribution (typically the 95th percentile) and the lower percentile of the fault distribution (typically the 5th percentile). This technique reduces the statistical overlap region between the classes and consequently decreases the probability of misclassification. Finally, the obtained values were evaluated through a cross-scenario validation process over the entire set of 8,100 simulated scenarios. At this stage, the performance of each threshold set was assessed based on evaluation metrics such as detection accuracy, false detection rate, and classification reliability.

The proposed protection algorithm was selected. Accordingly, the final values were determined as $Thr_{\text{1}}=\text{0}.\text{1}\text{0}$ p.u, $Thr_{\text{2}}=\text{0}.\text{1}\text{5}$, $Thr_{\text{3}}=\text{0}.\text{8}\text{5}$and $Thr_{\text{4}}=\text{0}.\text{9}\text{0}$ p.u. Here, $Thr_{\text{1}}$ and $Thr_{\text{4}}$are expressed per-unit because they are applied to normalized current and voltage indices, while $Thr_{\text{2}}$and $Thr_{\text{3}}$are dimensionless because they are used as lower and upper bounds of a relative current index. This way of expressing the thresholds is deliberately chosen in order to reduce the dependence of the decision values on the nominal voltage and current levels of a particular system. In other words, instead of using absolute thresholds in amperes or volts, the measured signals are first normalized to the base values of the same system and then compared with the proposed thresholds. Therefore, the reported thresholds are not limited to the numerical values of the simulated system and can be used for HVDC systems with different nominal levels, provided that the normalization process is performed based on the corresponding base values of the same system. In order to clarify the dimensional interpretation of the thresholds, the normalized differential current index, the current ratio index, and the normalized pole voltage indices are defined in Eq. (19), Eq. (20), and Eq. (21), respectively.


$$\begin{array}{c} I_{diff}^{pu}=\frac{\underset{t\in W}{\text{max}}|i_{diff}(t)|}{I_{base}} \end{array}$$ (19)

$$\begin{array}{c} R_{i}=\frac{\underset{t\in W}{\text{max}}|i_{com}(t)|}{\underset{t\in W}{\text{max}}|i_{diff}(t)|} \end{array}$$ (20)

$$\begin{array}{c} {\text{V}}_{T_{\text{1}}-p}^{pu}=\frac{\underset{t\in W}{\text{min}}|V_{T_{\text{1}}-p}|}{V_{base}},{\text{V}}_{T_{\text{1}}-n}^{pu}=\frac{\underset{t\in W}{\text{min}}|V_{T_{\text{1}}-n}|}{V_{base}} \end{array}$$ (21)

In the above relations, *W* is the decision time window after the fault occurs, $I_{diff}$ is the differential current, $i_{com}$ is the common current component, and $V_{T_{\text{1}}-p}$ and $V_{T_{\text{1}}-n}$ are the positive and negative pole voltages. According to Eq. (19), the threshold $Thr_{\text{1}}=\text{0}.\text{1}\text{0}$ p.u is the fault detection boundary based on the normalized differential current index. Therefore, its corresponding physical value in each system will be equal to $\text{0}.\text{1}\text{0}I_{base}$. As a result, whenever the numerical value of this index exceeds a fixed value, the fault is detected. According to Eq. (20), the index $R_{i}$is obtained by dividing two smooth current quantities and is therefore dimensionless. For this reason, this index does not depend on the absolute current level of the system and rather expresses the relative relationship between the current components under different fault conditions. The values $Thr_{\text{2}}=\text{0}.\text{1}\text{5}$ and $Thr_{\text{3}}=\text{0}.\text{8}\text{5}$ are used as the lower and upper bounds of this index in the decision-making logic. In other words, if the condition $\text{0}.\text{1}\text{5}\leq R_{i}\leq \text{0}.\text{8}\text{5}$ holds, the current condition corresponds to a single-pole-to-ground fault pattern. Since this criterion is defined based on the ratio of currents, its value is less affected by the system’s rated current scale and can be used in systems with different capacities and current levels. Similarly, the voltage indices from Eq. (21) are normalized to the base voltage. Therefore, the threshold $Thr_{\text{4}}=\text{0}.\text{9}\text{0}$ p.u. defines the detection boundary of a faulty pole and its corresponding physical value in each system will be $\text{0}.\text{9}\text{0}V_{\text{base}}$.

As a result, although the threshold value in per-unit space remains 0.90, its equivalent value in volts is determined based on the rated voltage of the system in question. Whenever the normalized voltage of a pole falls below this value during the decision time interval ($V_{p}^{\text{pu}}<\text{0}.\text{9}\text{0}\;\text{or}\;V_{n}^{\text{pu}}<\text{0}.\text{9}\text{0}$), that pole is considered as a pole involved in the fault. Thus, expressing the thresholds $Thr_{\text{4}}$ and $Thr_{\text{1}}$ in per-unit terms and expressing $Thr_{\text{3}}$ and $Thr_{\text{2}}$ in dimensionless terms has been done with the aim of increasing the generalizability of the algorithm and preventing its direct dependence on the absolute values of current and voltage of a specific system. This approach allows the proposed thresholds to be interpreted not as fixed values in amperes or volts, but as normalized decision boundaries that can be transferred to different HVDC systems. Therefore, if the algorithm is applied to another system, it is sufficient to normalize the current and voltage signals based on $I_{\text{base}}$ and $V_{\text{base}}$of the same system and then compare them with the above thresholds. Thus, the thresholds between $Thr_{\text{4}}$ and $Thr_{\text{1}}$ are per-unit and between $Thr_{\text{3}}$ and $Thr_{\text{2}}$ are dimensionless, with the aim of increasing the algorithm’s discriminability and avoiding its direct dependence on the absolute values of current and voltage of a specific system. This approach allows the proposed thresholds to be interpreted not as fixed values in amperes or volts, but as normalized decision boundaries that can be used in different HVDC systems.

Therefore, if the algorithm is applied to another system, it is sufficient to normalize the current and voltage signals based on $I_{\text{base}}$ and $V_{\text{base}}$of the same system and then compare them with the above thresholds. It is important to emphasize that these final values were not chosen empirically or based on trial and error, but were obtained through a decision boundary extraction process based on statistical analysis of the data distribution, including KDE probability density estimation, boundary refinement based on appropriate levels, and cross-scenario validation on a total of 8100 simulated cases. As a result, the selected thresholds strike a good balance between sensitivity to high-resistance faults and robustness to nonlinear transients, increasing the accuracy, reliability, and generalizability of the HVDC differential protection algorithm under different operating conditions and for systems with different rated levels.

## 3. Software simulation and results analysis

In Section 3, the detailed model of the simulated VSC–HVDC network implemented in MATLAB/Simulink is first introduced. The model includes the synchronous source, three-level converters, a bipolar DC link, and a DFIG-based wind farm. Subsequently, various PTG, NTG, and PTN fault scenarios with different locations, fault resistances, and fault inception times are simulated. The performance of the proposed algorithm in fault detection, classification, and location is then analyzed based on the voltage and current signals measured at terminal T_1_. Furthermore, eight sensitivity analysis studies are conducted to evaluate the robustness of the method against variations in operating conditions, fault resistance, DC line parameters, control strategies, DC link voltage level, load level, measurement uncertainties, signal delay, and filtering effects. The results of these studies are reported quantitatively and analytically within the section text, providing a clear assessment of the accuracy, stability, and reliability of the proposed protection algorithm under different operational conditions.

### 3.1 Introduction of the studied network in the MATLAB/simulink environment

**Fig 4:**
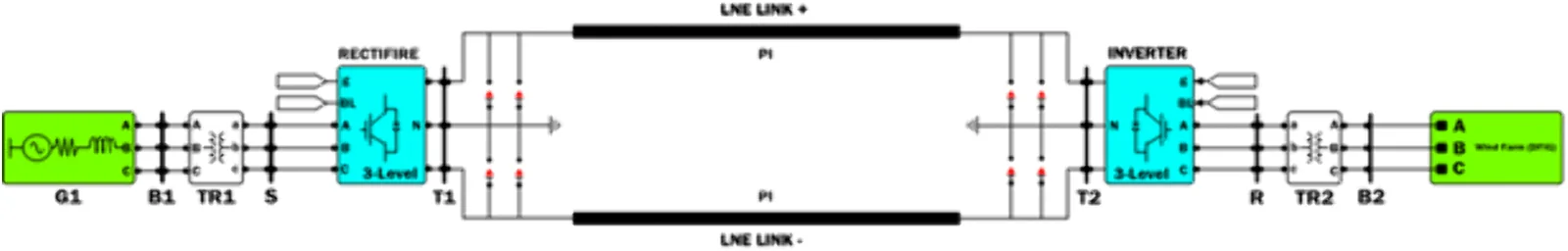
Diagram of the studied network implemented in the MATLAB Simulink environment.

Figure 4 illustrates the structure of the power network under study, which has been implemented in the MATLAB/Simulink environment. This system includes a two-terminal HVDC converter structure that transfers the power generated from a DFIG-based wind farm to the main grid. The purpose of this simulation is to analyze the dynamic performance of the system under various operating conditions and to investigate its response to potential disturbances on both the DC and AC sides. On the left side of the figure, the synchronous source $G_{\text{1}}$ is located, which represents the equivalent of the main AC grid. The rated power of this source is 320 MVA with a line-to-line voltage of 63 kV and a frequency of 50 Hz. The short-circuit ratio of this source is selected as 4, and its equivalent impedance includes a series resistance and inductance, each equal to 0.15 per unit, which models realistic grid conditions. Next, the generated power is transferred through a step-down transformer $TR_{\text{1}}$ from 230 kV to 63 kV and then applied to a three-level VSC converter. This converter is responsible for converting AC power to DC, and its structure is based on the three-level NPC topology. By employing appropriate AC filters, the output voltage quality is improved and major harmonic distortions are eliminated. The DC output of the converter is connected to the HVDC link. The middle section of the system consists of two positive and negative DC links with a nominal voltage of ±115 kV, responsible for power transmission over a distance of 300 km. Each pole has a resistance of R=0.015 Ω/km, an inductance of $L=\text{1}.\text{2}\times {\text{1}\text{0}}^{-\text{3}}$H/km, and a capacitance of $C=\text{0}.\text{0}\text{1}\text{2}\times {\text{1}\text{0}}^{-\text{6}}$F/km. To enhance the dynamic stability of the network, a smoothing reactor with a value of 150 mH and a filter capacitor with a capacitance of 80 µF are installed in the DC section. Furthermore, the measurement transducers are configured with a sampling frequency of 2 kHz to acquire the system signals. On the right side, the DC voltage and current are converted back to AC through another three-level converter referred to as the VSC inverter. This converter is connected to the wind farm bus through a step-up transformer $TR_{\text{2}}$ (from 33 kV to 63 kV). Both converters, including the rectifier and the inverter, have a rated power of 35 MW, a nominal voltage of ±115 kV, and a switching frequency of 1500 Hz, with each arm consisting of 200 submodules. The arm inductance is 50 mH and the arm resistance is considered to be 0.03 Ω. At the final section of the network, the wind farm is modeled as a DFIG system consisting of 50 wind turbines with a total power capacity of 20 MW. The voltage of the wind farm collection network is 63 kV and its operating frequency is 50 Hz. Overall, this simulation model represents an HVDC power transmission system with a VSC–HVDC structure between a synchronous source and a DFIG-based wind farm. This configuration is designed to study transient stability, active and reactive power control, and the network performance under various operating conditions. All subsystems, including the AC source, transformers, DC link, and wind farm, are parametrically configured according to Table 1 and are ready for comprehensive dynamic analysis in the Simulink environment.

**Table 1:** Parameters of the studied network.

G_1_ source		
**Variable**	**Value**	**Dimension**
Total rated power	320	MVA
Line-to-line RMS voltage	63	kV
Frequency	50	Hz
Short-circuit ratio	4	–
Equivalent source resistance	0.15	pu
Equivalent source inductance	0.15	pu
**Wind farm source (DFIG)**		
**Variable**	**Value**	**Dimension**
Total rated power	20	MW
AC collection grid voltage	63	kV
Frequency	50	Hz
Number of turbines	50	–
Equivalent R	0.05	pu
Equivalent L	0.12	pu
**HVDC Line (Positive & Negative Pole)**		
**Variable**	**Value**	**Dimension**
DC voltage rating	±115	kV
Number of poles	2	–
Line length	300	km
Tower spacing	400	m
Resistance per km	0.015	Ω/km
Inductance per km	1.2e−3	H/km
Capacitance per km	0.012e−6	F/km
**TR1 and TR2 transformers**		
**Variable**	**Value**	**Dimension**
Transformer type	Step-down Yg/Δ	–
Rated power	320	MVA
Primary voltage (HV)	230	kV
Secondary voltage (LV)	63	kV
Turns ratio	63/33 = 1.91	–
Short-circuit impedance	0.12	pu
Copper loss	0.002	pu
Core loss	0.001	pu
Magnetizing inductance	500	pu
**Rectifier & Inverter**		
**Variable**	**Value**	**Dimension**
Rated power	35	MW
Nominal DC voltage	±115 kV	kV
AC-side RMS voltage	33	kV
Switching frequency	1500	Hz
Number of submodules	200 per arm	–
Arm inductance	50	mH
Arm resistance	0.03	Ω
**DC link capacitance and resistance**		
**Variable**	**Value**	**Dimension**
DC smoothing reactor	150	mH
DC filter capacitance	80	µF
DC discharge resistance	150	kΩ
Initial DC voltage	115	kV

### 3.2 Simulation of various fault scenarios and interpretation of results

In this section, the performance of the proposed protection method is evaluated from two complementary perspectives. First, in Subsection 3.2.1, a set of different fault scenarios along the HVDC transmission line is simulated in order to investigate the behavior of the algorithm under diverse operating conditions in terms of fault type, fault distance, fault resistance, and fault inception time. In this part, the results obtained from the transient responses of current and voltage at the local terminal, as well as the outputs of the fault detection, classification, and location unit, are analyzed to demonstrate the accuracy and speed of the proposed method under various scenarios. Subsequently, in Subsection 3.2.2, a series of sensitivity analysis studies are conducted to examine the stability and robustness of the algorithm against variations in operating conditions and practical uncertainties such as changes in wind farm power level, fault resistance, line parameters, converter control strategies, DC link voltage level, AC network loading conditions, measurement noise, and signal delays. Together, these two subsections demonstrate that the proposed method not only performs accurately under different fault scenarios but also maintains reliable operational stability under various practical conditions of HVDC networks.

#### 3.2.1 Implementation of various fault scenarios.

**Fig 5:**
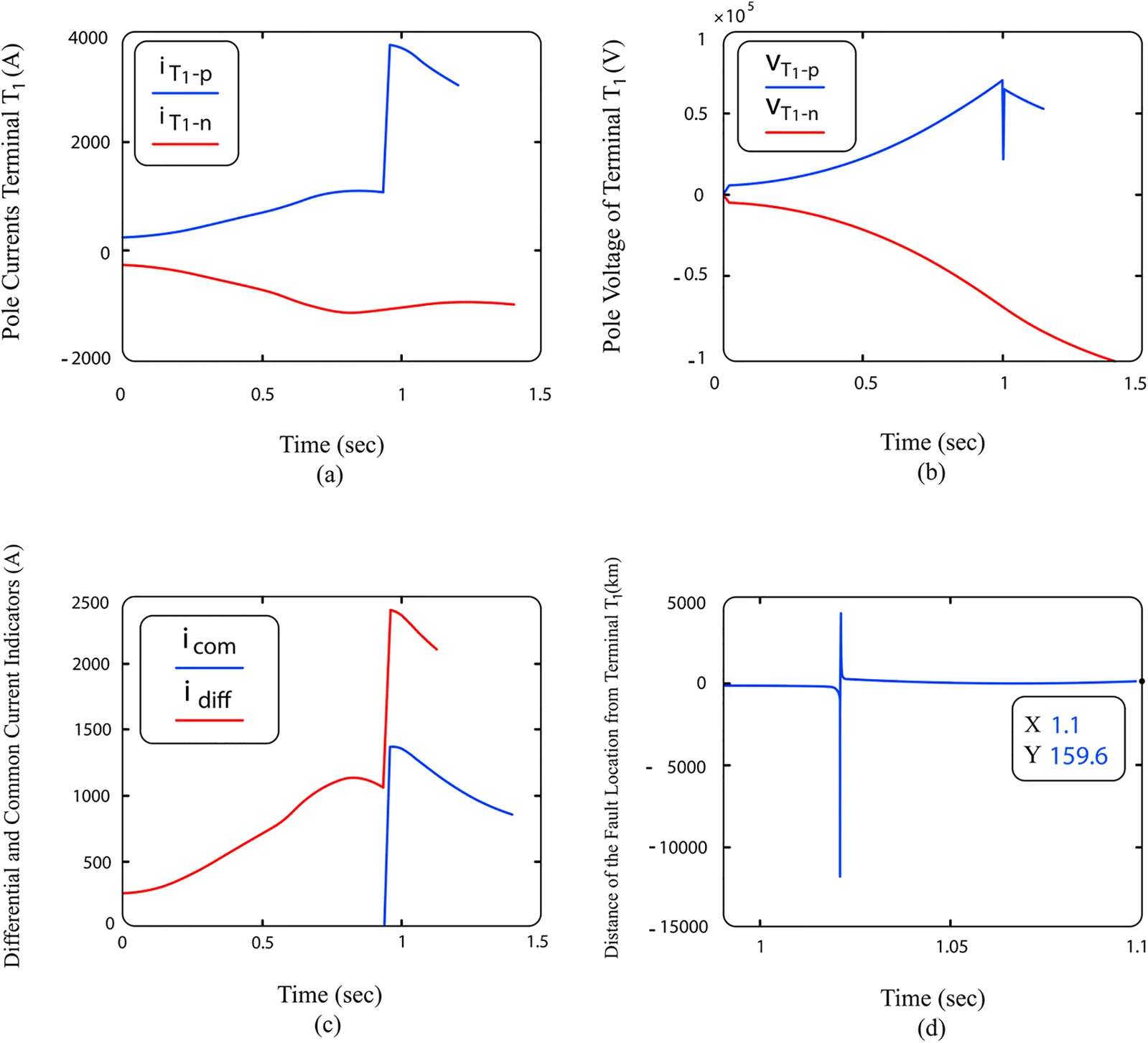
Results obtained from the implementation of Scenario 1. (a) Positive and negative pole currents measured at terminal $T_{\text{1}}$ in Scenario 1, (b) positive and negative pole voltages measured at terminal $T_{\text{1}}$ in Scenario 1, (c) differential and common currents of the positive and negative poles measured at terminal $T_{\text{1}}$ in Scenario 1, (d) estimated distance of the fault location relative to terminal $T_{\text{1}}$ in Scenario 1.

**Fig 6:**
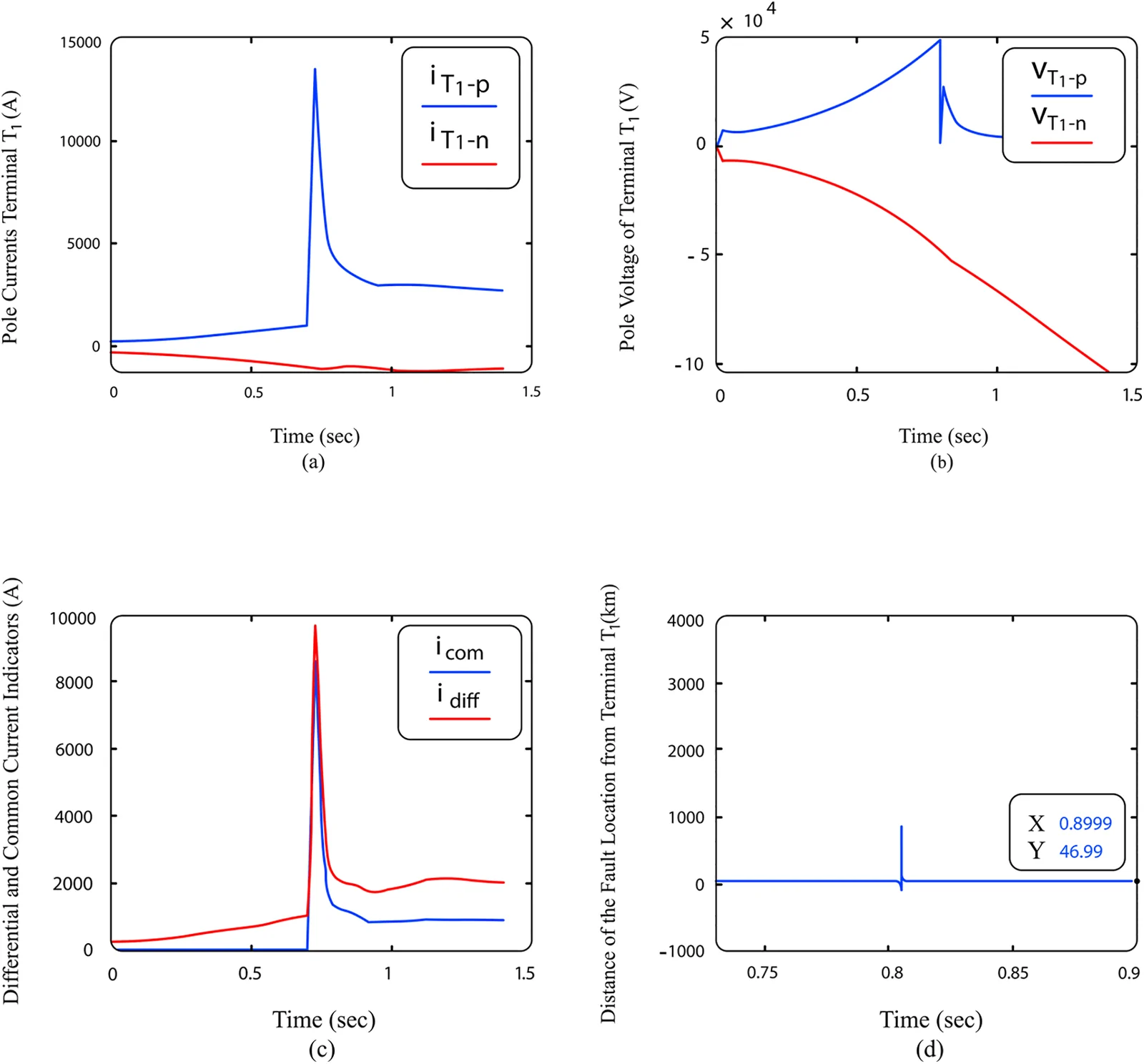
Results obtained from the implementation of Scenario 2. (a) Positive and negative pole currents measured at terminal $T_{\text{1}}$ in Scenario 2, (b) positive and negative pole voltages measured at terminal $T_{\text{1}}$ in Scenario 2, (c) differential and common currents of the positive and negative poles measured at terminal $T_{\text{1}}$ in Scenario 2, (d) estimated distance of the fault location relative to terminal $T_{\text{1}}$in Scenario 2.

**Fig 7:**
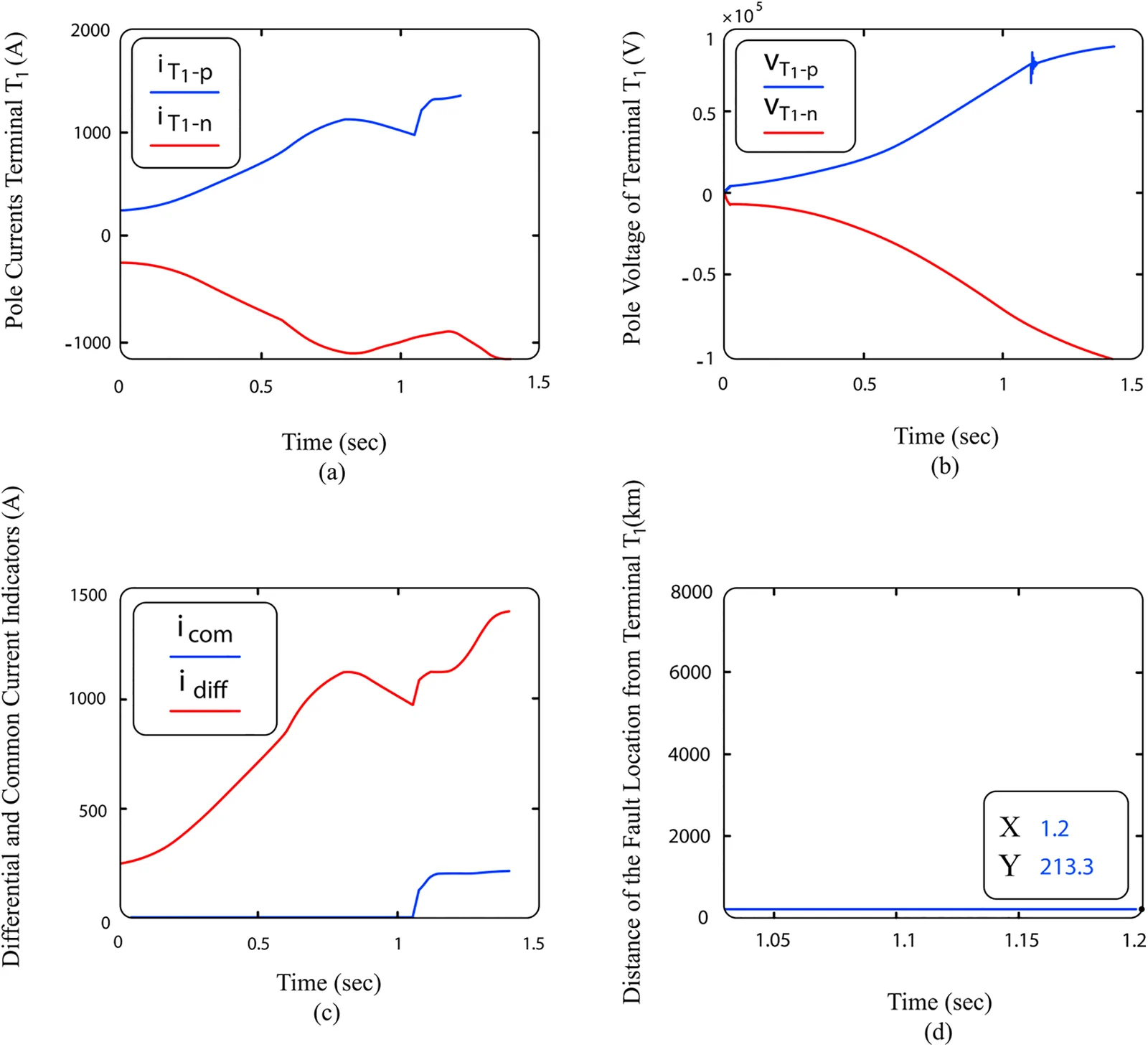
Results obtained from the implementation of Scenario 3. (a) Positive and negative pole currents measured at terminal $T_{\text{1}}$ in Scenario 3, (b) positive and negative pole voltages measured at terminal $T_{\text{1}}$ in Scenario 3, (c) differential and common currents of the positive and negative poles measured at terminal $T_{\text{1}}$ in Scenario 3, (d) estimated distance of the fault location relative to terminal $T_{\text{1}}$in Scenario 3.

**Fig 8:**
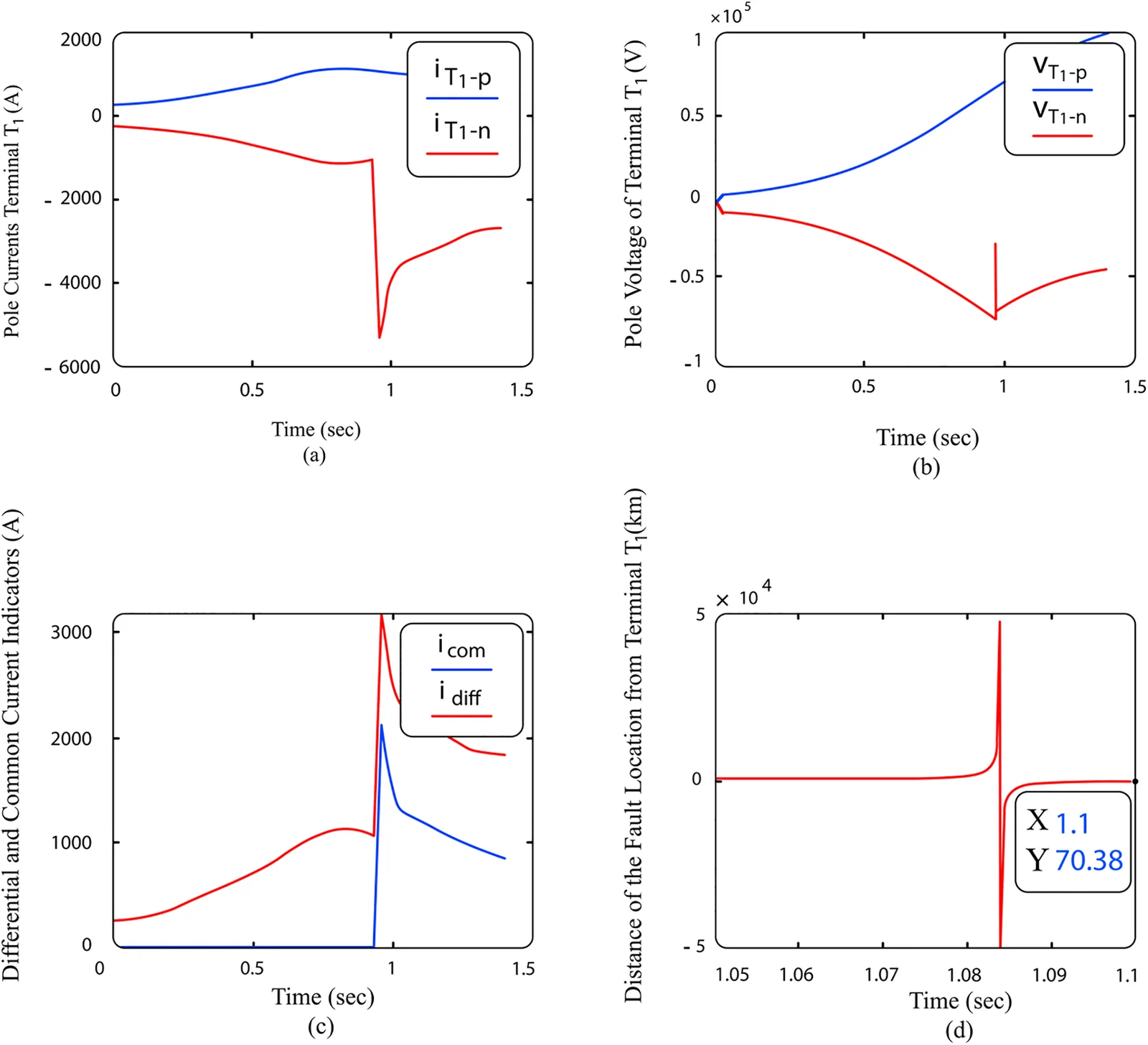
Results obtained from the implementation of Scenario 4. (a) Positive and negative pole currents measured at terminal $T_{\text{1}}$ in Scenario 4, (b) positive and negative pole voltages measured at terminal $T_{\text{1}}$ in Scenario 4, (c) differential and common currents of the positive and negative poles measured at terminal $T_{\text{1}}$ in Scenario 4, (d) estimated distance of the fault location relative to terminal $T_{\text{1}}$ in Scenario 4.

**Fig 9:**
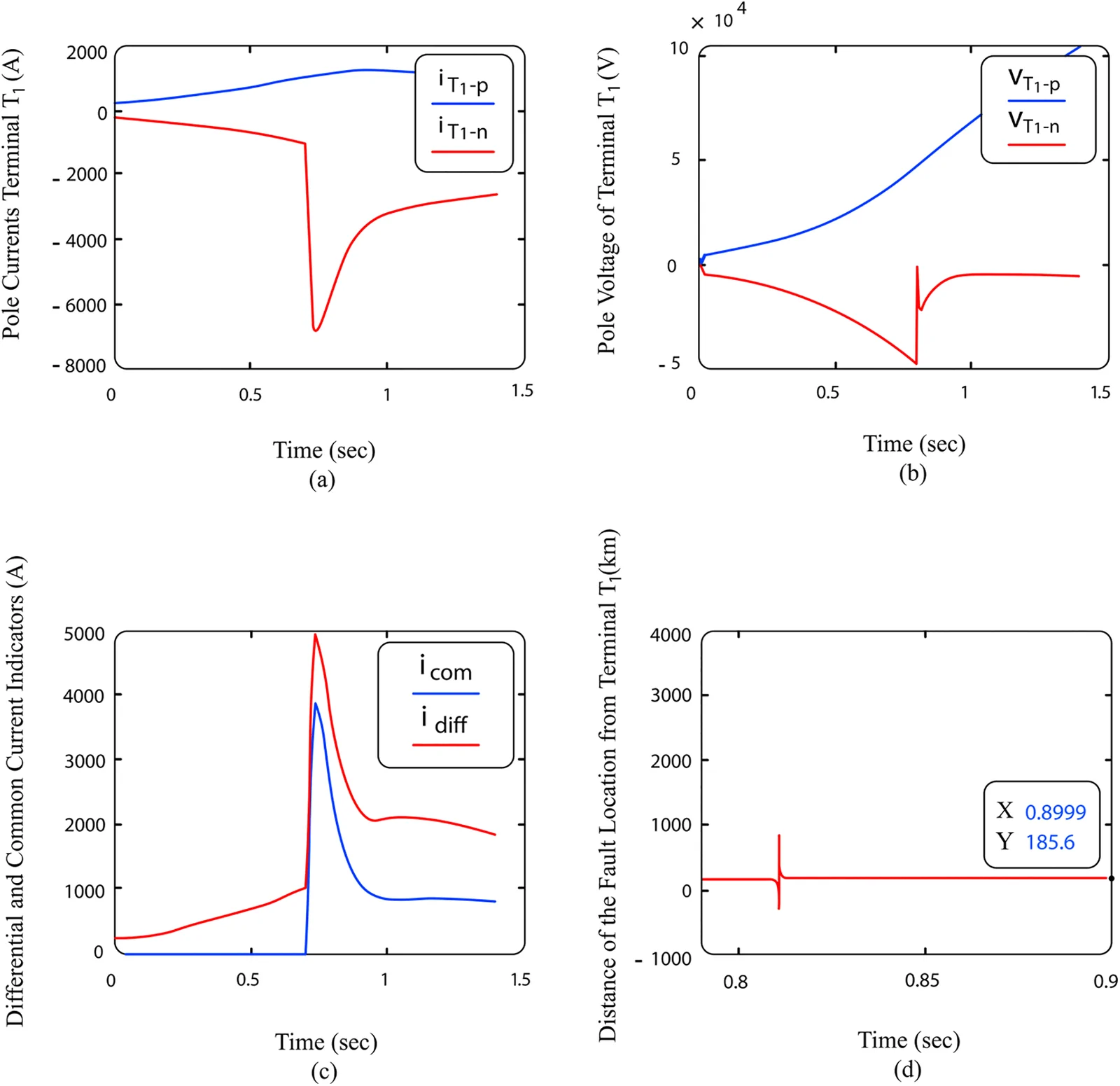
Results obtained from the implementation of Scenario 5. (a) Positive and negative pole currents measured at terminal $T_{\text{1}}$ in Scenario 5, (b) positive and negative pole voltages measured at terminal $T_{\text{1}}$ in Scenario 5, (c) differential and common currents of the positive and negative poles measured at terminal $T_{\text{1}}$ in Scenario 5, (d) estimated distance of the fault location relative to terminal $T_{\text{1}}$ in Scenario 5.

**Fig 10:**
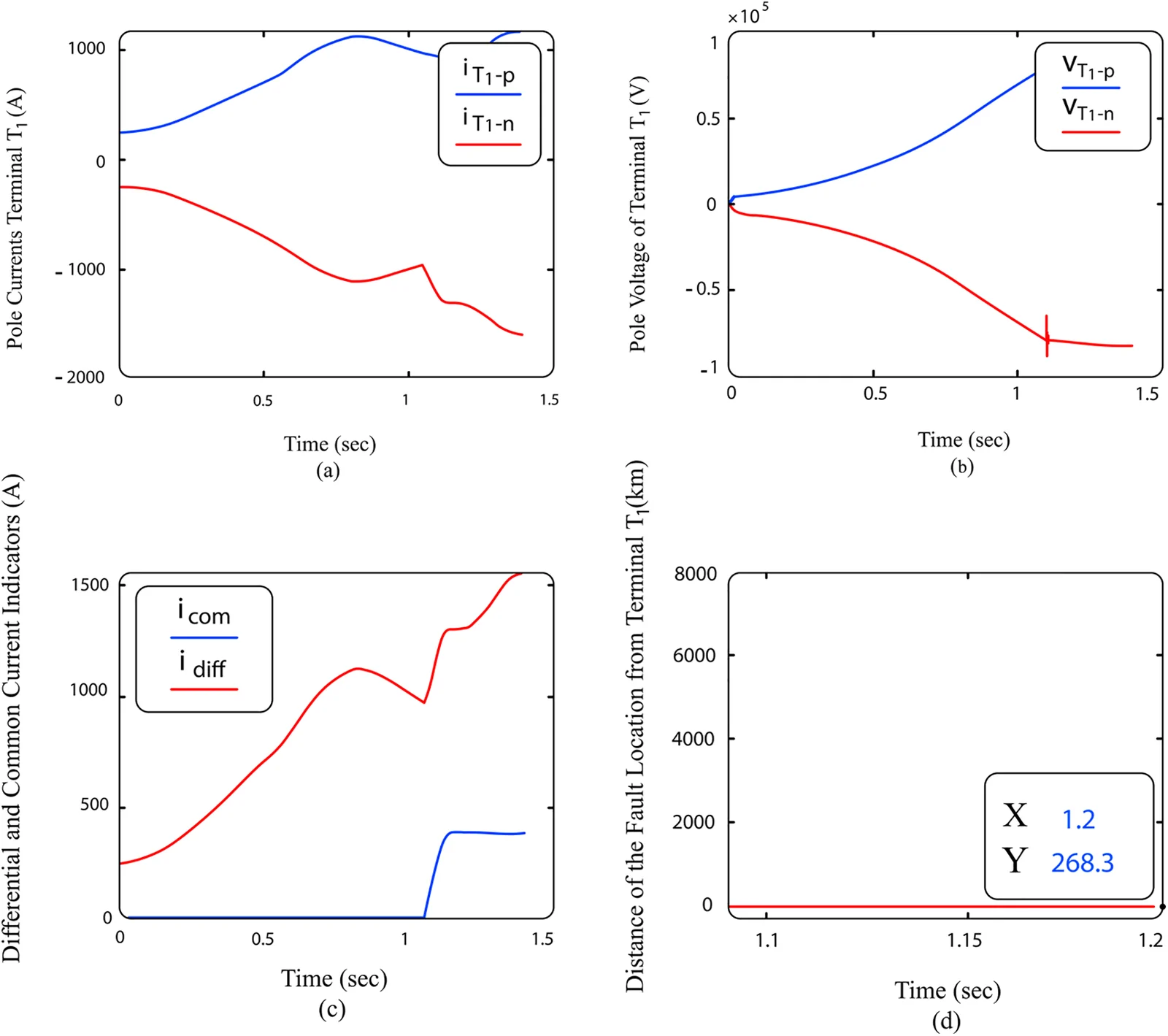
Results obtained from the implementation of Scenario 6. (a) Positive and negative pole currents measured at terminal $T_{\text{1}}$ in Scenario 6, (b) positive and negative pole voltages measured at terminal $T_{\text{1}}$ in Scenario 6, (c) differential and common currents of the positive and negative poles measured at terminal $T_{\text{1}}$ in Scenario 6, (d) estimated distance of the fault location relative to terminal $T_{\text{1}}$ in Scenario 6.

**Fig 11:**
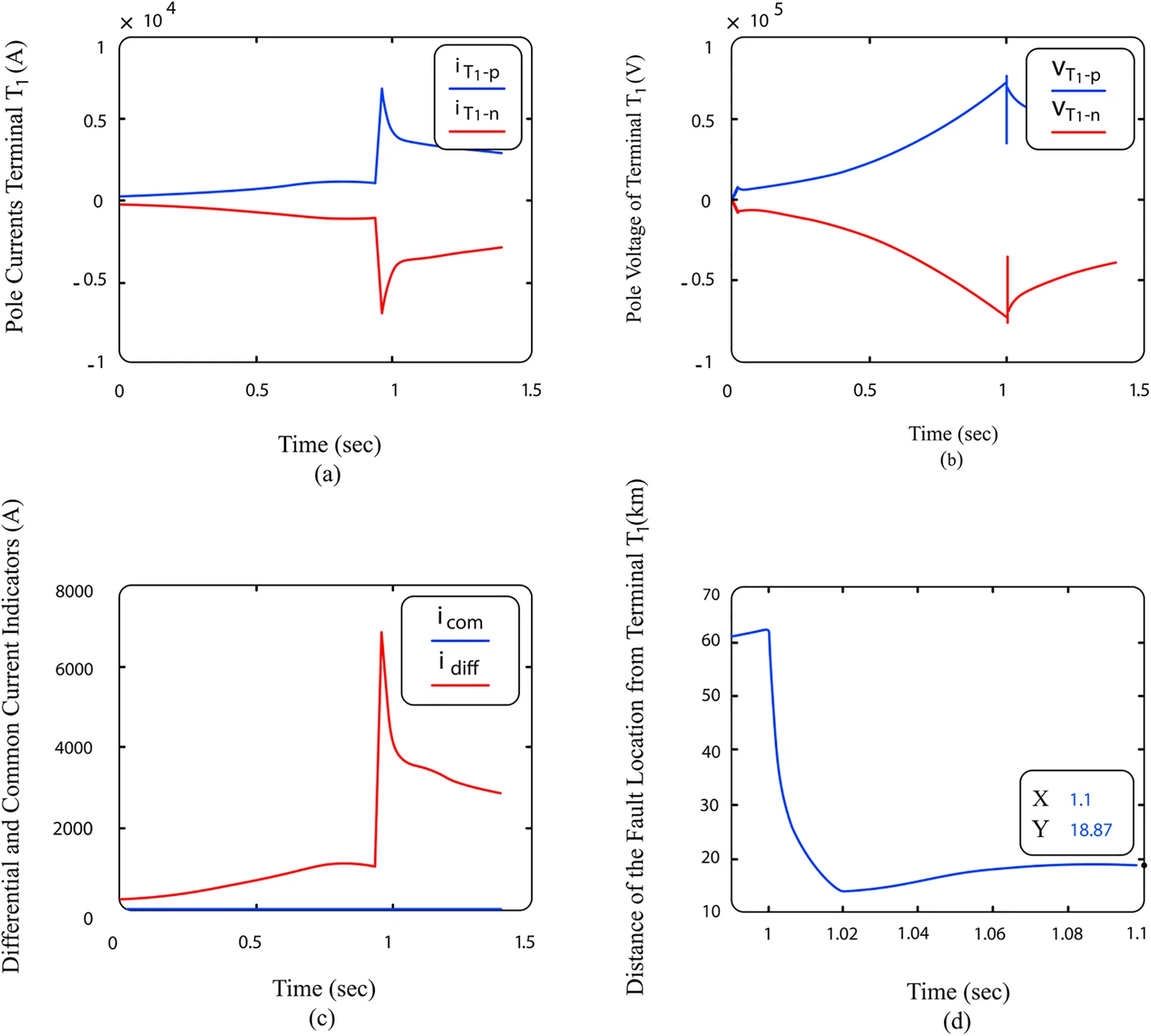
Results obtained from the implementation of Scenario 7. (a) Positive and negative pole currents measured at terminal $T_{\text{1}}$ in Scenario 7, (b) positive and negative pole voltages measured at terminal $T_{\text{1}}$ in Scenario 7, (c) differential and common currents of the positive and negative poles measured at terminal $T_{\text{1}}$ in Scenario 7, (d) estimated distance of the fault location relative to terminal $T_{\text{1}}$ in Scenario 7.

**Fig 12:**
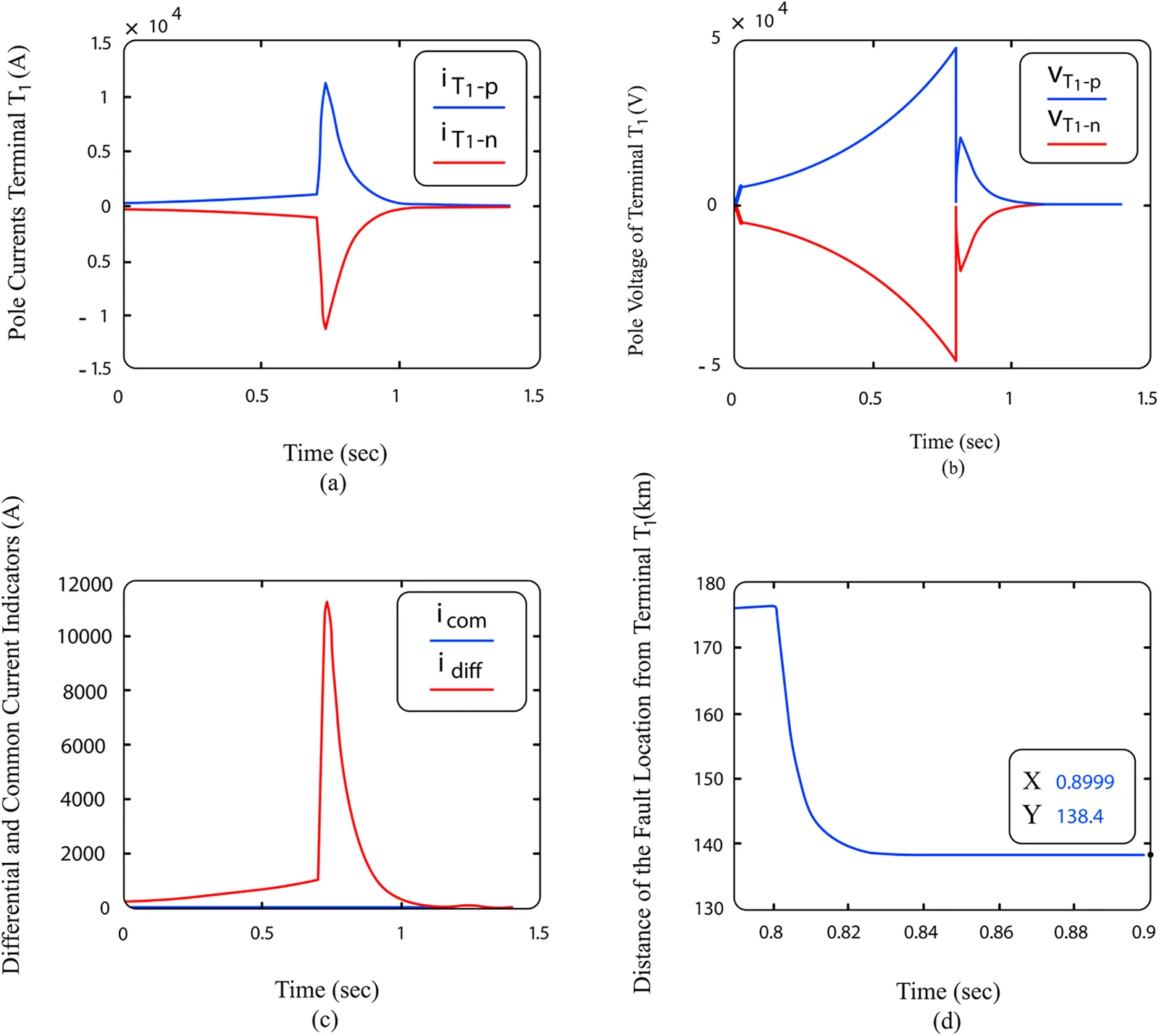
Results obtained from the implementation of Scenario 8. (a) Positive and negative pole currents measured at terminal $T_{\text{1}}$ in Scenario 8, (b) positive and negative pole voltages measured at terminal $T_{\text{1}}$ in Scenario 8, (c) differential and common currents of the positive and negative poles measured at terminal $T_{\text{1}}$ in Scenario 8, (d) estimated distance of the fault location relative to terminal $T_{\text{1}}$ in Scenario 8.

**Fig 13:**
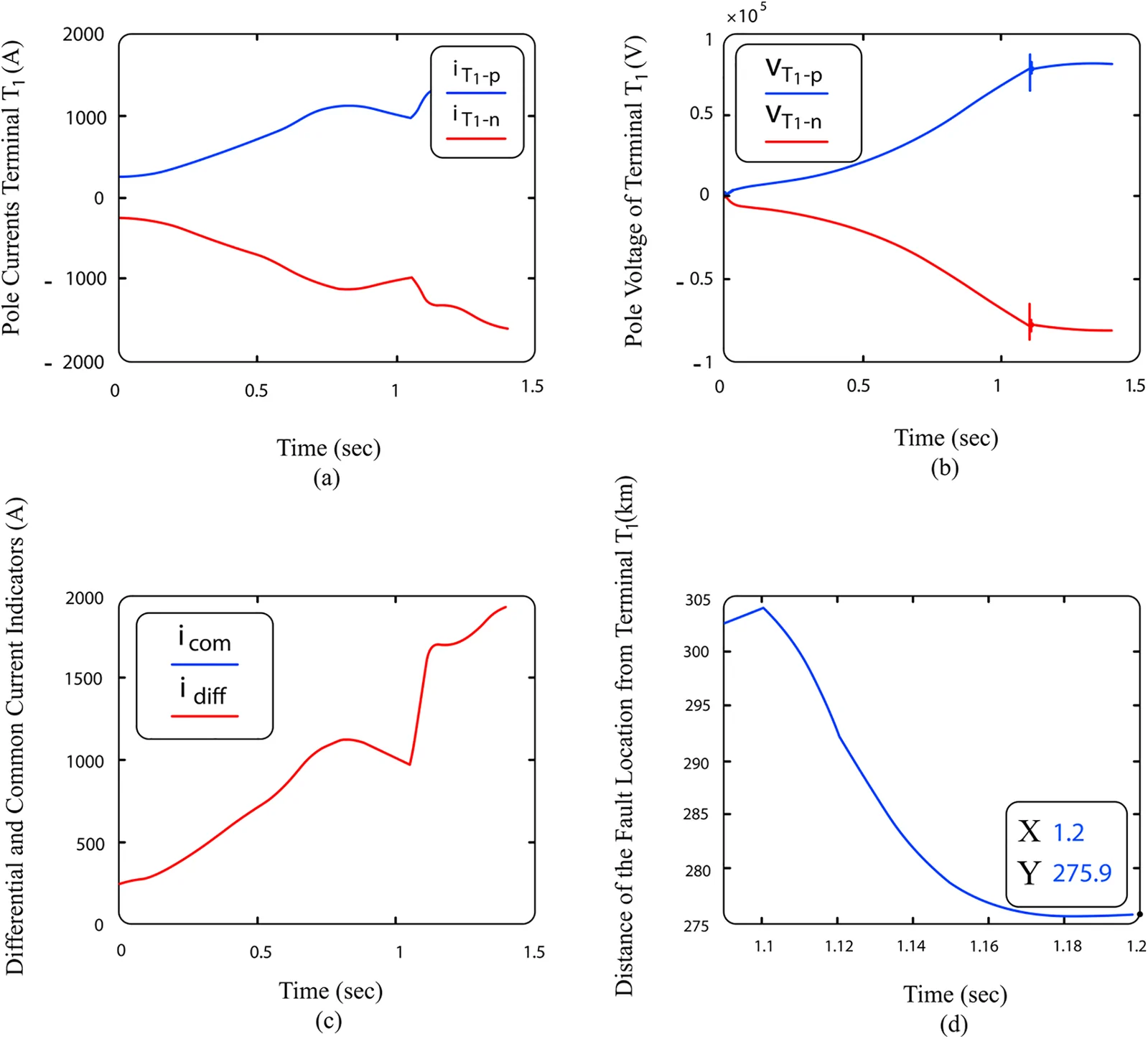
Results obtained from the implementation of Scenario 9. (a) Positive and negative pole currents measured at terminal $T_{\text{1}}$ in Scenario 9, (b) positive and negative pole voltages measured at terminal $T_{\text{1}}$ in Scenario 9, (c) differential and common currents of the positive and negative poles measured at terminal $T_{\text{1}}$ in Scenario 9, (d) estimated distance of the fault location relative to terminal $T_{\text{1}}$ in Scenario 9.

In this section, several diverse fault scenarios are examined to evaluate the performance of the proposed protection algorithm in the HVDC network. The objective of these simulations is to assess the capability of the method in detecting the occurrence of faults, classifying the fault type, and accurately locating the fault position under different operating and fault conditions. The selected scenarios include three main categories of faults, namely PTG, NTG, and PTN, each applied at different distances from terminal $T_{\text{1}}$, with varying fault resistances and different fault inception times. This configuration enables a comprehensive evaluation of the algorithm behavior under both near and distant faults, low-resistance and high-resistance faults, as well as different operating instants of the system. Specifically, in this set of simulations, the main parameters defining each scenario include the fault distance d, the fault resistance $R_{f}$, and the fault inception time. The fault resistance varies within the range of 1–100 Ω, while the fault inception time is considered as 0.8 s, 1 s, and 1.1 s in different cases. This diversity of parameters ensures that the algorithm is tested not only under simple and low-resistance conditions but also under more challenging scenarios involving higher fault resistance and longer distances from the terminal. Structurally, the results of this section are presented through several consecutive figures, each illustrating an independent scenario. According to the simulation results, Figs 5–13 correspond to different fault scenarios, where each figure illustrates the behavior of the measured variables and the algorithm output for a specific case. These measured variables include electrical quantities related to the current and voltage of the positive and negative poles at terminal $T_{\text{1}}$, which constitute the basis of the decision-making process in the fault detection, classification, and location stages. Therefore, the analysis of the results in this section shows that the proposed algorithm, based on these measured signals, is capable of correctly identifying the fault type and estimating the fault distance with acceptable accuracy. Overall, these scenarios are designed to demonstrate the stability, accuracy, and reliability of the proposed protection method, covering both near and far faults, low- and high-resistance faults, as well as all three types of monopolar and bipolar faults. Consequently, this section plays a crucial role in validating the practical effectiveness of the proposed algorithm under realistic HVDC network conditions.

***3.2.1.1. PTG*** *Faul**t at Distance d = 159*** ***km from Terminal T**_**1**_* ***with Fault Resistance R**_f_ **= 10 Ω*** ***at T_f_ = 1s.*** In the first scenario, the performance of the proposed protection algorithm is evaluated for a PTG fault occurring at a distance of 159 km from terminal T_1_, with a fault resistance of R_f_ = 10 ohm and a fault inception time of t_f_ = 1 s. The measured electrical quantities used for the analysis are illustrated in Fig 5. Figure 5(a) shows the positive and negative pole currents, Fig 5(b) presents the positive and negative pole voltages, Fig 5 (c) depicts the differential and common current indices calculated according to Eq. (1), and Fig 5(d) illustrates the estimated fault location obtained using Eq. (16).

As shown in Fig 5(a), the occurrence of the fault leads to a sudden transient increase in the positive pole current. Based on these measurements, the indices i_diff_ and i_com_ are calculated according to Eq. (1), and their behavior is presented in Fig 5(c). Immediately after the fault inception, the index idiff exceeds the threshold Thr1, while the ratio max(i_com_)/max(i_diff_) remains within the interval [Thr_1_, Thr_2_]. According to the detection logic presented in the flowchart of Fig 2, this behavior confirms the occurrence of a PTG fault. The detection delay is approximately 2.3 ms, indicating the fast response capability of the proposed method.

The fault classification stage is based on the voltage behavior illustrated in Fig 5(b). A significant drop is observed in the positive pole voltage V_T1-p_, which crosses the threshold Thr_4_, while the negative pole voltage V_T1-n_ remains nearly unchanged. This asymmetric voltage behavior between the two poles satisfies the classification criteria and correctly identifies the fault type as PTG.

Finally, the fault location is determined using Eq. (16), and the resulting location index is shown in Fig 5(d). After the initial transient oscillations, the estimated location converges to approximately 159.6 km, which is very close to the actual fault distance. The corresponding location error is less than 0.2 percent, demonstrating the high accuracy of the proposed algorithm in fault detection, classification, and fault location under the operating conditions of this scenario.

***3.2.1.2. PTG Fault at Distance d = 159 km from Terminal T_1_ with Fault Resistance R_f_ = 1 Ω* *at T_f_ = 0.8s.*** In the second scenario, the proposed protection algorithm is evaluated for a PTG fault occurring at a distance of 47 km from terminal T_1_, with a fault resistance of R_f_ = 1 ohm and a fault inception time of t_f_ = 0.8 s. The measured signals and calculated indices are illustrated in Fig 6. Figure 6(a) shows the positive and negative pole currents, Fig 6(b) presents the corresponding pole voltages, Fig 6 (c) depicts the differential and common current indices obtained from Eq. (1), and Fig 6(d) shows the estimated fault location obtained using Eq. (16). As shown in Fig 6(a), the fault occurrence produces a sharp rise in the positive pole current immediately after t_f_. Using these measurements, the indices i_diff_ and icom are calculated according to Eq. (1), and their behavior is presented in Fig 6 (c). Following the fault inception, i_diff_ exceeds the threshold Thr_1_, while the ratio max(i_com_)/ max(i_diff_) remains within the interval [Thr_1_, Thr_2_]. According to the detection logic of the protection algorithm, this response confirms the occurrence of a PTG fault. The fault is detected with a delay of approximately 3.65 ms.

The voltage behavior illustrated in Fig 6(b) further supports the classification stage. A pronounced drop is observed in the positive pole voltage V_T1-p_ after t_f_, causing it to fall below the threshold Thr_4_, whereas the negative pole voltage V_T1-n_ remains nearly unchanged. This asymmetric voltage response between the poles satisfies the classification conditions and correctly identifies the fault type as PTG.

The estimated fault location obtained using Eq. (16) is presented in Fig 6(d). After the initial transient response, the location index converges to approximately 47 km, which closely matches the actual fault position. The location error, calculated based on the absolute difference between the estimated and actual distances and normalized with respect to the line length, is less than 0.0033 percent. These results confirm that the proposed algorithm maintains high accuracy in detection, classification, and location even under low fault resistance conditions and for faults occurring closer to the terminal.

***3.2.1.3. PTG Fault at Distance d = 214 km from Terminal T_1_ with Fault Resistance R_f_ = 100 Ω* *at T_f_ = 1.1s.*** In this scenario, the proposed protection algorithm is evaluated for a PTG fault occurring at a distance of 214 km from terminal T_1_, with a relatively high fault resistance of R_f_ = 100 ohm and a fault inception time of t_f_ = 1.1 s. The measured signals and calculated indices are presented in Fig 7. Figure 7(a) shows the positive and negative pole currents, Fig 7(b) illustrates the corresponding pole voltages, Fig 7 (c) depicts the differential and common current indices obtained from Eq. (1), and Fig 7(d) shows the estimated fault location obtained using Eq. (16). As shown in Fig 7(a), the high fault resistance limits the magnitude of the fault current compared with the previous scenarios. Nevertheless, immediately after t_f_, the positive pole current still exhibits a clear transient variation. Based on these current measurements, idiff and icom are calculated according to Eq. (1), and their behavior is shown in Fig 7 (c). After the fault inception, idiff exceeds the threshold Thr_1_, while the ratio max(i_com_)/ max(i_diff_) remains within the interval [Thr_1_, Thr_2_]. According to the detection logic of the proposed algorithm, this response confirms the occurrence of a PTG fault. The fault is detected with a delay of approximately 2.69 ms, showing that the algorithm maintains a fast response even under high-resistance fault conditions. The fault classification stage is supported by the voltage behavior presented in Fig 7(b). After t_f_, the positive pole voltage V_T1-p_ decreases and crosses the threshold Thr_4_, whereas the negative pole voltage V_T1-n_ remains nearly unchanged. Although the voltage drop is less severe than in low-resistance cases, the asymmetric voltage response between the two poles remains sufficiently distinguishable. Therefore, the classification criterion correctly identifies the fault type as PTG. Finally, the fault location result obtained using Eq. (16) is shown in Fig 7(d). Due to the combined effect of high fault resistance and the relatively long distance from terminal T_1_, the location index shows a slightly smoother convergence compared with the previous scenarios. However, after the initial transient interval, the estimated location stabilizes at approximately 213.3 km, which is close to the actual fault distance of 214 km. The corresponding location estimation error, calculated based on the absolute difference between the estimated and actual fault distances and expressed as a percentage of the line length, is approximately 0.233 percent. This result confirms that the proposed algorithm can accurately detect, classify, and locate PTG faults even when the fault current and voltage variations are weakened by high fault resistance.

***3.2.1.4. NTG Fault at Distance d = 71 km from Terminal T_1_ with Fault Resistance R_f_ = 10 Ω* *at T_f_ = 1s.*** In the fourth scenario, the proposed protection algorithm is evaluated for an NTG fault occurring at a distance of 71 km from terminal T_1_, with a fault resistance of R_f_ = 10 ohm and a fault inception time of t_f_ = 1 s. The measured signals and calculated indices are presented in Fig 8. Figure 8(a) shows the positive and negative pole currents, Fig 8(b) illustrates the corresponding pole voltages, Fig 8(c) depicts the differential and common current indices calculated according to Eq. (1), and Fig 8(d) shows the estimated fault location obtained using Eq. (17). As shown in Fig 8(a), the fault occurrence produces a clear transient variation in the pole currents immediately after t_f_. Based on these current measurements, i_diff_ and icom are calculated using Eq. (1), as illustrated in Fig 8 (c). After the fault inception, idiff exceeds the threshold Thr_1_, while the ratio max(i_com_)/ max(i_diff_) remains within the interval [Thr_1_, Thr_2_]. According to the detection logic of the proposed algorithm, this response confirms the occurrence of an NTG fault. The fault is detected with a delay of approximately 2.99 ms, indicating the fast response of the protection scheme for negative pole faults. The fault classification stage is supported by the voltage behavior shown in Fig 8(b). After tf, the negative pole voltage V_T1-n_ experiences a significant drop and crosses the threshold Thr_4_, whereas the positive pole voltage V_T1-p_ remains nearly unchanged. This asymmetric voltage variation is consistent with the expected behavior of an NTG fault and satisfies the classification criterion. Therefore, the proposed algorithm correctly identifies the fault type as NTG. Finally, the fault location result obtained using Eq. (18) is shown in Fig 8(d). After the initial transient interval, the location index converges to approximately 70.38 km, which is very close to the actual fault distance of 71 km. The corresponding location estimation error, calculated based on the absolute difference between the estimated and actual fault distances and expressed as a percentage of the line length, is approximately 0.207 percent. These results confirm that the proposed algorithm can accurately detect, classify, and locate NTG faults with moderate fault resistance, while maintaining a performance level comparable to that obtained for positive pole faults.

***3.2.1.5. NTG Fault at Distance d = 186 km from Terminal T_1_ with Fault Resistance R_f_ = 1 Ω* *at T_f_ = 0.8s.*** In the fifth scenario, the proposed protection algorithm is examined for an NTG fault occurring at a distance of 186 km from terminal T_1_, with a low fault resistance of R_f_ = 1 ohm and a fault inception time of t_f_ = 0.8 s. The corresponding current and voltage responses, as well as the calculated indices and location results, are illustrated in Fig 9. Figure 9(a) presents the positive and negative pole currents, Fig 9(b) shows the pole voltages, Fig 9 (c) depicts the differential and common current indices obtained using Eq. (1), and Fig 9(d) illustrates the estimated fault location calculated using Eq. (17).

As observed in Fig 9(a), the occurrence of the fault produces a pronounced transient surge in the negative pole current immediately after tf, which is characteristic of a low-impedance fault connected to the negative pole. This rapid current variation results in a significant increase in the differential current component. Accordingly, the index idiff calculated from Eq. (1) quickly exceeds the threshold Thr_1_, while the ratio max(i_com_)/ max(i_diff_) remains within the interval [Thr_1_, Thr_2_], as shown in Fig 9 (c). Based on the detection logic of the proposed protection scheme, this response confirms the presence of a monopolar ground fault. The detection delay in this scenario is approximately 2.86 ms, indicating that the algorithm preserves a fast detection capability even for faults occurring far from the measuring terminal.

The classification stage is validated by the voltage responses depicted in Fig 9(b). At the instant of the fault, the negative pole voltage V_T1-n_ experiences a sharp drop and crosses the threshold Thr_4_, reflecting the strong electrical coupling between the fault point and the negative pole. In contrast, the positive pole voltage V_T1-p_ remains nearly unaffected and does not cross the threshold, exhibiting only a minor transient disturbance caused by system coupling. This clear voltage asymmetry between the poles satisfies the classification conditions and enables the algorithm to correctly identify the fault type as NTG.

The fault location estimation obtained using Eq. (17) is shown in Fig 9(d). Following the initial transient period, the location index gradually stabilizes as the measured quantities approach steady conditions. The estimated fault position converges to approximately 185.6 km from terminal T_1_, which is very close to the actual distance of 186 km. The corresponding location estimation error, calculated based on the absolute difference between the estimated and actual fault distances and expressed as a percentage of the line length, is approximately 0.133 percent. These results demonstrate that the proposed algorithm maintains a high level of accuracy in fault location even for remote NTG faults with very low fault resistance, confirming the robustness and reliability of the method under diverse operating conditions.

***3.2.1.6. NTG Fault at Distance d = 269 km from Terminal T_1_ with Fault Resistance R_f_ = 100 Ω* *at T_f_ = 1.1s.*** In the sixth scenario, the proposed protection scheme is evaluated under a challenging NTG fault condition located at a long distance of 269 km from terminal T_1_ with a relatively high fault resistance of R_f_ = 100 Ω occurring at t_f_ = 1.1 s. The corresponding electrical responses and calculated indices are illustrated in Fig 10. Specifically, Fig 10(a) shows the positive and negative pole currents, Fig 10(b) presents the pole voltages, Fig 10 (c) illustrates the differential and common current indices obtained using Eq. (1), and Fig 10(d) depicts the fault location estimated based on Eq. (17).

As shown in Fig 10(a), the high resistance of the fault limits the magnitude of the resulting fault current; however, a distinct transient variation can still be observed in the negative pole current immediately after the fault inception. This disturbance produces a measurable increase in the differential current component. Consequently, the calculated index i_diff_ rapidly exceeds the threshold Thr_1_, while the ratio max(i_com_)/ max(i_diff_) remains within the interval [Thr_1_, Thr_2_], as illustrated in Fig 10 (c). According to the detection logic of the proposed method, these conditions confirm the occurrence of a monopolar ground fault. Despite the relatively weak current magnitude caused by the high fault resistance and long electrical distance, the detection module successfully identifies the fault with a delay of approximately 3.22 ms, demonstrating the sensitivity of the algorithm to high-impedance faults.

Further validation of the fault type is obtained from the voltage responses shown in Fig 10(b). At the fault inception instant, the negative pole voltage V_T1-n_ exhibits a noticeable drop and crosses the threshold Thr_4_, whereas the positive pole voltage V_T1-p_ remains largely unaffected except for minor transient disturbances caused by electromagnetic coupling between the poles. This asymmetric voltage behavior clearly satisfies the classification criteria of the proposed protection strategy and leads to the correct identification of the fault as an NTG event.

The performance of the location module is illustrated in Fig 10(d). After the transient components decay, the variables involved in the location formulation stabilize, allowing the algorithm to compute a reliable estimate of the fault position using Eq. (17). The estimated distance converges to approximately 268.3 km from terminal T_1_. Compared with the actual fault distance of 269 km, the calculated location error is approximately 0.23%, obtained from the absolute difference between the estimated and actual distances expressed as a percentage of the line length. The obtained accuracy confirms that the proposed method maintains stable fault detection, reliable classification, and precise location capability even under unfavorable operating conditions.

***3.2.1.7. PTN Fault at Distance d = 19 km from Terminal T_1_ with Fault Resistance R_f_ = 10 Ω* *at T_f_ = 1 s*.** In this scenario, the performance of the proposed protection algorithm is evaluated for a PTN fault occurring at a distance of 19 km from terminal T_1_, with a fault resistance of R_f_ = 10 Ω and a fault inception time of t_f_ = 1 s. The corresponding current and voltage responses, calculated indices, and location result are illustrated in Fig 11. Figure 11(a) presents the positive and negative pole currents, Fig 11(b) shows the pole voltages, Fig 11(c) depicts the differential and common current indices obtained using Eq. (1), and Fig 11(d) illustrates the estimated fault location calculated using Eq. (18).

As observed in Fig 11(a), the occurrence of the bipolar fault produces a simultaneous disturbance in both pole currents. Unlike monopolar faults, where the disturbance is mainly concentrated in one pole, the PTN fault involves both conductors in the fault current path. This results in a pronounced and nearly synchronous increase in the positive and negative pole currents. Consequently, the differential current index i_diff_ calculated using Eq. (1) rapidly exceeds the threshold Thr_1_, as shown in Fig 11(c). Based on the detection criteria of the proposed algorithm, the fault is detected within approximately 3.66 ms, demonstrating the fast response of the method for severe bipolar faults.

The classification stage is mainly supported by the voltage behavior shown in Fig 11(b). Immediately after the fault inception, both pole voltages V_T1-p_ and V_T1-n_ experience significant drops and cross the threshold Thr_4_. This simultaneous voltage collapse in both poles represents a distinctive electrical signature of PTN faults, since both conductors are directly involved in the fault path. Therefore, the concurrent threshold crossing satisfies the classification logic and enables the algorithm to clearly identify the fault type as PTN.

Following the detection and classification stages, the fault location procedure is performed using Eq. (18). As illustrated in Fig 11(d), the location output converges rapidly after the initial transient interval and stabilizes at approximately 18.87 km from terminal T_1_. Compared with the actual fault distance of 19 km, the absolute location deviation is 0.13 km. Accordingly, the location estimation error is approximately 0.043%, calculated from the absolute difference between the estimated and actual distances and expressed as a percentage of the line length. This low error confirms the high accuracy of the proposed method in locating near-terminal PTN faults.

Overall, the results of this scenario demonstrate that the proposed algorithm can effectively detect, classify, and locate PTN faults with high accuracy and rapid response. The strong and simultaneous electrical signatures produced by the bipolar fault enable the protection scheme to achieve fast detection and stable location convergence even when the fault occurs close to the measuring terminal.

***3.2.1.8. PTN Fault at Distance d = 139 km from Terminal T_1_ with Fault Resistance R_f_ = 1 Ω* *at T_f_ = 0.8 s.*** In this scenario, the proposed protection algorithm is evaluated for a PTN fault occurring at a distance of 139 km from terminal T1, with a low fault resistance of R_f_ = 1 Ω and a fault inception time of **t**_**f**_ = **0.8 s**. The corresponding responses are illustrated in Fig 12, where Fig 12(a) shows the positive and negative pole currents, Figure 12(b) presents the pole voltages, Fig 12 (c) depicts the differential and common current indices obtained using Eq. (1), and Fig 12(d) shows the estimated fault location calculated using Eq. (18).

As shown in Fig 12(a), the fault produces a strong and simultaneous rise in both pole currents immediately after inception, which is consistent with the electrical behavior of a low-resistance bipolar fault. This disturbance causes the differential current index **i**_**diff**_ to increase rapidly and exceed the threshold Thr_1_, while the associated fault criteria are satisfied in the first instants following the disturbance, as illustrated in Fig 12(c). This enables the proposed algorithm to detect the fault with a fast response; specifically, the detection delay in this scenario is 2.69 ms. The classification stage is confirmed by the voltage waveforms in Fig 12(b). At the fault instant, both **V**_**T1-p**_ and **V**_**T1-n**_ undergo sharp drops and cross the threshold Thr_4_ almost simultaneously. Since both poles are directly involved in the fault path, this concurrent voltage collapse represents a clear signature of a PTN fault. Therefore, the classification logic correctly identifies the fault type as PTN. The fault location result is presented in Fig 12(d). After the short transient interval, the location estimator converges to approximately 138.4 km from terminal T_1_. Compared with the actual fault distance of 139 km, the absolute deviation is 0.6 km, corresponding to a location estimation error of approximately 0.20%, calculated from the absolute difference between the estimated and actual distances expressed as a percentage of the line length. These results confirm that the proposed method maintains accurate and stable performance for distant PTN faults even under low-resistance conditions.

**3.2.1.9. *PTN Fault at Distance d = 276 km from Terminal T_1_ with Fault Resistance R_f_ = 100 Ω* *at T_f_ = 1.1 s.*** In this scenario, the performance of the proposed protection algorithm is evaluated for a distant PTN fault occurring at d = 276 km from terminal T_1_ with a relatively high fault resistance of R_f_ = 100 Ω at tf = 1.1 s. The corresponding responses are illustrated in Fig 13, where Fig 13(a) presents the positive and negative pole currents, Fig 13(b) shows the pole voltages, Fig 13 (c) depicts the differential and common current indices obtained using Eq. (1), and Fig 13(d) illustrates the estimated fault location calculated using Eq. (18). As shown in Fig 13(a), the occurrence of the bipolar fault produces noticeable disturbances in the currents of both poles despite the relatively high fault resistance and the long electrical distance from the measuring terminal. This disturbance leads to a clear increase in the differential current index i_diff_, which rapidly exceeds the threshold Thr_1_, as illustrated in Fig 13 (c). According to the detection logic of the proposed method, these conditions confirm the presence of a fault. The detection stage is completed with a delay of approximately 3.02 ms, indicating that the algorithm maintains fast response characteristics even under high-impedance fault conditions. The classification stage is verified using the voltage responses shown in Fig 13(b). Immediately after the fault inception, both pole voltages V_T1-p_ and V_T1-n_ experience simultaneous drops and cross the threshold Thr_4_. This concurrent voltage collapse in both conductors represents the typical electrical signature of PTN faults, since both poles are directly involved in the fault path. Consequently, the classification logic of the proposed protection scheme correctly identifies the fault type as PTN. The performance of the location module is presented in Fig 13(d). After the transient components decay, the variables used in the location formulation reach steady conditions, allowing the fault position to be estimated using Eq. (18). The location output converges to 275.9 km, yielding a relative error of 0.033%. This indicates that the proposed method provides an accurate estimation that closely matches the actual fault distance. This result confirms that the proposed algorithm maintains stable convergence and reliable accuracy even for distant PTN faults with relatively high fault resistance, demonstrating its robustness for HVDC transmission line protection.

The numerical results and transient waveforms illustrated in Figs 5 – 13 confirm the analytical integrity of the proposed protection scheme under diverse operating conditions. A detailed examination of the current indices *i*_*diff*_ and *i*_*com*_ in the detection stage reveals that the magnitude of the initial peak and the settling time are highly sensitive to the fault location and resistance. For instance, in close-up faults with low resistance (e.g., Fig 6), the sharp gradient of the differential index *i*_*diff*_*/dt* allows for almost instantaneous threshold crossing, whereas in scenarios involving high resistance (100 Ω) or long distances (e.g., Figs 10 and 13), the wavefront suffers from dispersion. This dispersion, caused by the line’s frequency-dependent losses, reduces the high-frequency content of the fault signal, explaining the slight increase in detection delays (up to 3.66 ms). Numerically, this correlates with the damping factor of the HVDC system, where the fault resistance *R*_*f*_ acts as a dissipative element that absorbs the energy of the initial traveling wave, thereby smoothing the transient peak. Furthermore, the fault location trajectories shown in the figures demonstrate high stability after the initial transient oscillations subside. The location error, which remains remarkably low (below 0.24%), is a function of the accuracy of the per-unit-length voltage drop estimation. In PTG and NTG scenarios (Figs 5 – 11), the minor fluctuations in the estimated distance *d_est* during the first few milliseconds are due to the capacitive discharging currents of the line, which introduce a transient offset in Eq. (16) and (17). However, as shown in the PTN scenario results (Fig 13), the averaging approach in Eq. (18) significantly enhances the numerical precision. By processing both pole voltages simultaneously, the algorithm effectively filters out the mutual coupling noise and the induced voltages from the healthy sections of the bipolar configuration. This explains why Scenario 9, despite being at the far end of the line (276 km), yields an error of only 0.033%. These numerical outcomes validate that the proposed method effectively compensates for the non-linear voltage distribution along the line, ensuring reliable performance even when the signal-to-noise ratio is degraded by high fault impedance.

#### 3.2.2 Sensitivity analysis of the proposed method.

To comprehensively evaluate the robustness, reliability, and generalization capability of the proposed protection algorithm, a set of sensitivity analyses has been conducted under various fault conditions, network parameters, measurement quality levels, and HVDC system operating states. In HVDC transmission systems, the transient behavior of voltage and current following a fault can be influenced by several factors, including fault resistance, noise and distortion in measurement signals, DC-link voltage level, transmission line length, electrical parameters of the line, AC system strength, variations in renewable generation, converter operating conditions, and network topology. These factors may significantly affect the performance of protection algorithms; therefore, investigating the sensitivity of the proposed method to such variations is essential for assessing its practical applicability.

In this study, the system parameters and operating conditions were systematically varied from their nominal values in a controlled manner, and the performance of the algorithm in fault detection, classification, and location estimation was thoroughly examined. In each scenario, the fault detection delay, the accuracy of identifying the fault type and the involved pole, and the precision of fault location estimation were evaluated as the main performance indicators. It should be noted that, in all these investigations, the threshold values of the algorithm were kept unchanged without any retuning. The use of normalized and dimensionless indices ensures that the decision boundaries of the algorithm remain sufficiently stable against variations in system parameters and operating conditions.

To provide a comprehensive performance evaluation of the proposed method, eleven different sensitivity analysis scenarios have been considered. These include: analysis of the algorithm performance under high-impedance faults; investigation of the impact of CT saturation; assessment of algorithm robustness in the presence of noise in measured voltage and current signals; sensitivity analysis of the thresholds with respect to variations in the HVDC transmission line voltage level; evaluation of the impact of transmission line length variation; sensitivity analysis with respect to changes in transmission line parameters; investigation of the effect of AC system strength variation; evaluation of algorithm performance under sudden changes in wind speed and wind farm power output; assessment of the method in larger HVDC networks with multi-terminal topologies; sensitivity analysis with respect to variations in converter operating conditions and control modes; and finally, evaluation of the algorithm performance under DC-link voltage deviation.

**Fig 14:**
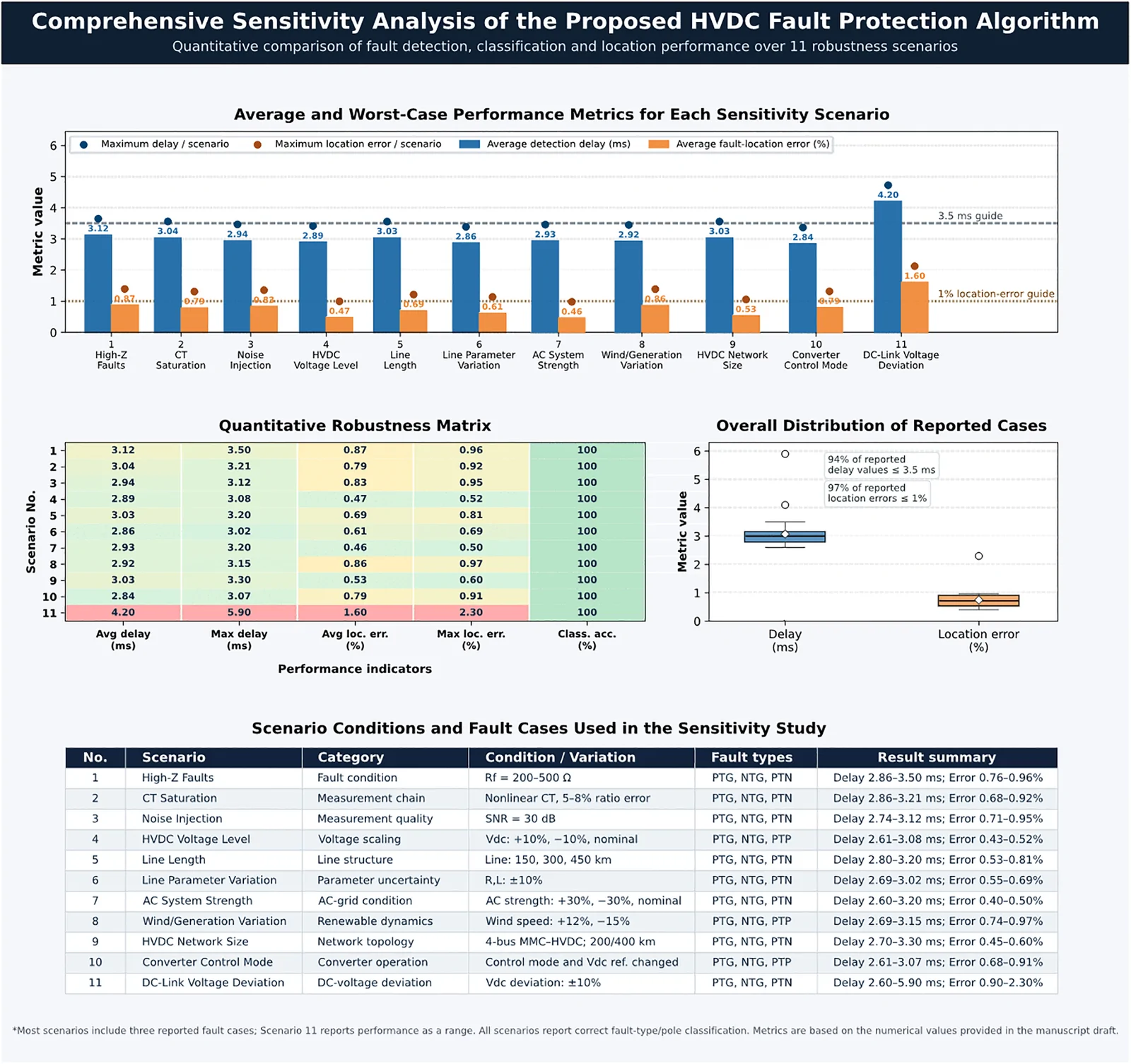
Infographic analysis of the results obtained from implementing the proposed algorithm under critical conditions.

At the end of these eleven sensitivity analysis scenarios, an infographic summarizing and analyzing the obtained results is presented in Fig 14.

***3.2.2.1 Sensitivity analysis with respect to high-impedance faults.*** To assess the robustness of the proposed algorithm under high-impedance fault conditions, a series of simulations was conducted considering relatively large values of fault resistance. In this scenario, three fault types, namely PTG, NTG, and PTN, were applied at distances of 80, 180, and 280 km from terminal T_1_, respectively. The corresponding fault resistances were set to 500, 200, and 300 Ω, while the fault inception times were selected as 1.2, 0.8, and 1.0 s, respectively. These resistance values were intentionally selected to create challenging conditions for the proposed algorithm, since high-impedance faults considerably attenuate the transient components of the measured signals. As a result, fault detection and analysis may become less accurate for many protection schemes. At the fault detection stage, the results show that the proposed algorithm can identify the faults with only a very short delay relative to the actual fault inception time. The detection delays for the three investigated cases were 3.5, 2.86, and 3.01 ms, respectively. Achieving such short detection times, despite the attenuation of transient signals caused by high fault resistance, indicates that the indices used in the algorithm have sufficient sensitivity and fault-detection capability. For fault classification and faulty-pole identification, the algorithm also demonstrates highly reliable performance. In all three cases, the faulty pole was correctly identified. This finding confirms that the decision criteria employed in the proposed algorithm provide adequate discrimination capability and can accurately distinguish the fault type and the involved pole, even in the presence of large fault resistances. Finally, the fault location errors for the three cases were 0.96%, 0.76%, and 0.89%, respectively, all below 1%. These results indicate that a significant increase in fault resistance does not noticeably degrade the fault-location performance of the proposed algorithm. In other words, the features extracted by the proposed method remain stable and relatively insensitive to the fault resistance value. Overall, the results demonstrate that the proposed algorithm maintains fast fault detection, correct faulty-pole classification, and accurate fault location even under high-impedance fault conditions, which are among the most challenging cases for protection systems. This confirms the robustness and reliability of the proposed method under practical operating conditions. It should also be noted that the behavior of the system under arcing faults or nonlinear fault impedances may be considered as an independent research topic in future work.

***3.2.2.2 Sensitivity analysis with respect to CT saturation.*** To evaluate the performance of the proposed algorithm under CT saturation conditions, a nonlinear CT model was included in the current measurement path. In this model, the core magnetization characteristic was represented by a piecewise nonlinear B-H curve, with the knee point located at approximately 1.2 pu of the rated flux [24,25]. Before the knee point, the effective core slope was assumed to be constant, whereas beyond this point, the magnetizing inductance decreases sharply. Following the occurrence of a fault at t = 1.2 s with a fault resistance of 1 ohm, the fault current rises to approximately 8–10 pu of the rated current within the first few milliseconds. At the instant of fault inception, due to the fault inception angle with respect to the voltage waveform, a DC offset component of about 0.3 pu of the AC component amplitude appears in the current. This component biases the core flux. As a result, the core flux exceeds 1 pu after approximately 3–4 ms from the fault inception and reaches the range of 1.2–1.3 pu, causing the CT to enter the saturation region. In the saturation region, the magnetizing inductance decreases to less than 20–30% of its linear value, while the magnetizing current increases to approximately 10–15% of the secondary current. This phenomenon leads to clipping at the peaks of the secondary current waveform and reduces the amplitude of the fundamental component. Accordingly, the equivalent measurement model experiences a ratio error of about 5–8% and a phase error of approximately 1–2 degrees during the first few half-cycles after the fault. The saturated portion of the waveform typically exhibits the highest distortion around 6–10 ms after fault inception, after which the distortion gradually decreases as the DC component decays. Under these conditions, three fault types, namely PTG, NTG, and PTN, were applied at distances of 15, 150, and 285 km from terminal T_1_, respectively, at t = 1.2 s. Despite the distortion introduced into the current signal, the proposed algorithm successfully detects the faults with delays of 2.86, 3.04, and 3.21 ms, respectively. It also correctly identifies the fault type and the involved pole in all three cases. Moreover, the fault location errors for these scenarios are 0.68%, 0.76%, and 0.92%, respectively, confirming the satisfactory robustness of the proposed algorithm against CT saturation-induced distortions.

***3.2.2.3 Sensitivity analysis with respect to noise injection into the current and voltage measurements at terminal T***_***1***_**.** To evaluate the stability of the proposed algorithm under measurement uncertainty, the effect of noise injection into the current and voltage measurements obtained from terminal T_1_ was investigated. In this scenario, noise was added numerically to the four measured signals at the T_1_ bus, namely the positive-pole voltage, positive-pole current, negative-pole voltage, and negative-pole current. Therefore, the noise was not injected only into the current or only into the voltage signals; rather, it was simultaneously applied to both the current and voltage signals of the positive and negative DC links.

The injected noise was modeled as zero-mean Gaussian white noise. For the numerical implementation, the RMS value of each measured signal was first calculated over the pre-fault interval, and the noise amplitude was then determined based on the corresponding RMS value [24,25]. In this study, a noise level corresponding to a signal-to-noise ratio (SNR) of 30 dB was selected. This is equivalent to an effective noise value of approximately 3.16% of the RMS value of the original signal. In other words, if the RMS value of the current or voltage in one pole is equal to a given value, the injected noise for that signal has an RMS value of about 0.0316 times the RMS value of the same signal. This procedure was applied independently to the positive-pole voltage, positive-pole current, negative-pole voltage, and negative-pole current. From a time-domain perspective, the noise was injected into the measured signals from t = 0.98 s, i.e., 20 ms before the fault inception, and continued until the end of the simulation window. Since the fault inception time in this scenario was set to t = 1.0 s, the algorithm was evaluated using noisy data in both the pre-fault and post-fault intervals. This choice makes the simulation conditions closer to a practical measurement system, where measurement noise is inherently present in voltage and current signals and does not appear only after fault inception. In the numerical implementation, an independent random noise sequence was generated for each of the four measured signals at terminal T_1_. These sequences followed a Gaussian distribution, had zero mean, and corresponded to an SNR of 30 dB. Each noise sequence was then added to the time-domain samples of its associated original signal. Consequently, at each sampling instant, the final input to the algorithm consisted of the true signal value plus a small random component. This procedure introduces random fluctuations into the voltage and current signal amplitudes and degrades the quality of the algorithm input data in a controlled manner. In this scenario, three fault types, namely PTG, NTG, and PTN, were applied at distances of 80, 180, and 280 km from terminal T_1_, respectively. The fault inception time was set to t = 1.0 s for all cases, and the fault resistance was considered to be 100 ohms in each case. Thus, the algorithm was assessed under conditions in which its input signals were contaminated by measurement noise both before and after fault inception. The obtained results show that the proposed algorithm maintains stable performance even in the presence of noise in the measured voltage and current signals of both the positive and negative links at the T_1_ bus. Despite the noise-induced distortion, the faults were detected with delays of 2.74, 2.96, and 3.12 ms, respectively, indicating only a limited and acceptable increase in the decision-making time. This confirms that the features extracted by the algorithm are sufficiently robust against random signal disturbances. Moreover, at the fault classification stage, the fault type and the involved pole were correctly identified in all three scenarios, and the algorithm successfully distinguished among PTG, NTG, and PTN faults. In the fault location stage, the distance estimation errors for the three cases were 0.71%, 0.83%, and 0.95%, respectively. These results indicate that noise injection has only a limited impact on the final accuracy of the proposed algorithm. Overall, this sensitivity analysis demonstrates that the proposed algorithm is sufficiently robust against noise in the current and voltage measurements obtained from terminal T_1_. Although noise with a specified intensity was simultaneously applied to the voltage and current signals of both the positive and negative poles, the algorithm was still able to perform fault detection, classification, and location estimation with acceptable accuracy.

***3.2.2.4 Sensitivity analysis of threshold values with respect to the HVDC transmission-line voltage level.*** To examine the dependence of the proposed algorithm’s threshold values on the voltage level of the HVDC transmission line, the effects of increasing and decreasing the DC voltage relative to its nominal value were evaluated. Since variations in the voltage level can change the absolute values of voltage and current in the system, protection methods that define their thresholds based on absolute quantities may experience a shift in their decision boundaries. In contrast, the thresholds in the proposed algorithm are defined using normalized and dimensionless indices; therefore, their performance is expected to remain stable against changes in the system voltage level. In the proposed algorithm, the decision thresholds are defined as Thr_1_ = 0.10 p.u., Thr_2_ = 0.15, Thr_3_ = 0.85, and Thr_4_ = 0.90 p.u. Thresholds Thr_1_ and Thr_4_ are associated with the differential current and pole-voltage indices in the per-unit domain, whereas Thr_2_ and Thr_3_ are used as limits for the current-ratio index and are dimensionless in nature. Accordingly, the decision-making process of the algorithm is not governed by the absolute scale of voltage and current, but by quantities normalized with respect to the system base values.

To evaluate this feature, the DC-link voltage level was changed before fault inception so that the system entered the fault condition at a specified operating voltage. In the first scenario, the DC voltage was increased by 10% at t = 0.45 s, and a PTG fault was applied at t = 0.5 s, 90 km from terminal T_1_. In the second scenario, the DC voltage was decreased by 10% at t = 0.65 s, and an NTG fault was simulated at t = 0.7 s, 190 km from terminal T_1_. To provide a reference case for comparison, the voltage level was kept at its nominal value in the third scenario, and a PTP fault was applied at t = 0.9 s, 290 km from terminal T_1_. In all cases, the fault resistance was set to 100 ohms.

The simulation results show that changing the HVDC line voltage level does not have a significant impact on the performance of the decision thresholds. Under the 10% voltage-increase condition, the PTG fault was detected with a delay of 2.61 ms, and the fault location was estimated close to the actual point, with an error of approximately 0.52% of the line length. Under the voltage-decrease condition, the NTG fault was detected with a delay of 2.98 ms, and the corresponding fault-location error was about 0.47% of the line length. In the nominal-voltage case, the PTP fault was detected with a delay of 3.08 ms, and the algorithm estimated the fault location with an approximate error of 0.43% of the line length.

In all scenarios, the thresholds associated with the current-ratio index and pole-voltage indices also correctly identified the fault type and the involved pole. These results indicate that, although variations in the voltage level change the amplitudes of the measured signals, the decision boundaries of the algorithm remain unchanged due to signal normalization with respect to the base values and the use of dimensionless indices. Consequently, the proposed threshold values do not have a direct dependence on the nominal voltage level of the system and can be applied to HVDC systems with different voltage ratings. This characteristic enhances the generalization capability and operational robustness of the proposed algorithm under various operating conditions.

***3.2.2.5 Sensitivity analysis of threshold values with respect to the HVDC transmission-line length.*** To investigate the dependence of the proposed algorithm’s threshold values on the HVDC transmission-line length, the performance of the protection method was evaluated under different line-length conditions. Variations in line length can affect the transient characteristics of the system, the propagation time of travelling waves, the attenuation level of the measured signals, and the wave-reflection pattern at the line terminals. Therefore, in some protection methods where the decision thresholds are defined based on absolute signal values or are tuned according to the physical parameters of the line, changing the line length may degrade the accuracy of fault detection, classification, or location estimation. In contrast, the proposed algorithm defines its thresholds based on normalized and dimensionless indices; hence, the line length is not expected to have a significant impact on the performance of these thresholds.

In the proposed algorithm, the decision thresholds are defined as Thr_1_ = 0.10 p.u., Thr_2_ = 0.15, Thr_3_ = 0.85, and Thr_4_ = 0.90 p.u. Thresholds Thr_1_ and Thr_4_ are derived from normalized signals in the per-unit domain, whereas Thr_2_ and Thr_3_ correspond to the current-ratio index and are dimensionless. Accordingly, these thresholds are not dependent on the absolute value of the line length; rather, the decision process is governed by the relative and normalized behavior of the measured signals. This characteristic provides the proposed threshold settings with suitable stability against structural variations in the network, including changes in the transmission-line length. To evaluate this aspect, the HVDC transmission-line length was examined under three different conditions. In the first case, the line length was increased by 50% relative to its nominal value. Assuming a nominal length of 300 km, the line length in this case was set to 450 km. In this scenario, a PTG fault was applied at t = 0.75 s at a distance of 335 km from terminal T_1_. The proposed algorithm detected the fault at t = 0.7528 s, corresponding to a detection delay of 2.8 ms with respect to the fault inception time. The classification stage also operated correctly, and the fault was accurately identified as a PTG fault. Moreover, the estimated fault distance was 336.1 km, which corresponds to a location error of approximately 0.81% of the line length. In the second case, the line length was reduced by 50% relative to the nominal value; therefore, the line length was considered to be 150 km. In this scenario, an NTG fault was applied at t = 1.5 s at a distance of 90 km from terminal T_1_, corresponding to 60% of the line length. The algorithm detected the fault at t = 1.5031 s, indicating a detection delay of 3.1 ms. In this case, the fault classification stage also performed correctly, and the fault type was accurately identified as NTG. The estimated fault distance was 88.9 km, corresponding to a location error of approximately 0.73% of the line length. In the third case, the line length was kept at its nominal value of 300 km to provide a reference condition for comparison. Under this condition, a PTN fault was applied at t = 1.8 s at a distance of 240 km from terminal T_1_, equivalent to 80% of the line length. The proposed algorithm detected the fault at t = 1.8032 s, corresponding to a detection delay of 3.2 ms. In this scenario, the classification stage again operated correctly, and the fault was accurately identified as a PTN fault. The estimated fault distance was 238.4 km, which corresponds to a location error of approximately 0.53% of the line length. The simulation results show that, despite considerable variations in the transmission-line length, the proposed threshold values remain capable of ensuring fast fault detection, correct fault classification, and accurate fault location estimation. In the case of a 50% increase in line length, although the wave propagation time and transient signal characteristics are modified, the normalized indices of the algorithm still cross the decision boundaries, allowing the fault to be detected with high accuracy. Similarly, in the case of a reduced line length, despite the shorter wave propagation path, the decision thresholds maintain satisfactory performance without requiring any retuning. The results also show that the fault-location error remains below approximately 1% of the line length in all investigated scenarios.

Overall, these results indicate that variations in the HVDC transmission-line length do not have a significant effect on the performance of the proposed algorithm’s decision thresholds. Owing to the use of normalized indices and dimensionless decision boundaries, the thresholds Thr_1_ = 0.10 p.u., Thr_2_ = 0.15, Thr_3_ = 0.85, and Thr_4_ = 0.90 p.u. can be applied to HVDC lines with different lengths without retuning. This feature confirms the stability and generalization capability of the proposed protection algorithm under different operating conditions.

***3.2.2.6 Sensitivity analysis with respect to transmission-line parameter variations.*** To evaluate the robustness of the proposed algorithm against uncertainty in the HVDC transmission-line parameters, a scenario was considered in which three fault types, namely PTG, NTG, and PTN, were applied at t = 1.0 s with a fault resistance of 100 ohms. To investigate the effect of line-parameter variations, the line resistance and inductance were modified in the three cases. Specifically, the resistance and inductance were increased by 10% in the first case, decreased by 10% in the second case, and increased by 10% in the third case. These variations were introduced to represent practical uncertainties, such as errors in line-parameter estimation, temperature-dependent changes in conductor characteristics, and inaccuracies in system modeling. At the fault detection stage, the proposed algorithm exhibited fast and stable performance. The detection delays with respect to the actual fault inception time were 2.69, 3.02, and 2.88 ms for the three investigated cases, respectively. These values show that the applied variations in the transmission-line parameters do not have a noticeable effect on the fault detection speed, and that the algorithm is able to identify fault inception within a very short time. For fault classification and faulty-pole identification, the proposed method also demonstrated fully reliable performance. In all three scenarios, both the fault type and the involved pole were correctly identified. This indicates that the decision criteria employed in the algorithm have adequate stability against network-parameter variations, and that relative changes in the line resistance and inductance do not disturb the fault-type identification process. Finally, the fault-location results show that the distance estimation errors for the three scenarios were 0.60%, 0.55%, and 0.69%, respectively. These values confirm the high accuracy of the fault-location stage even in the presence of transmission-line parameter variations. Overall, the results of this scenario demonstrate that the proposed algorithm has considerable robustness and stability against uncertainty and variations in the transmission-line parameters. It can maintain fast fault detection, correct classification, and accurate fault location estimation with high precision. This feature is particularly important from a practical application perspective, since transmission-line parameters in real systems are always subject to some degree of uncertainty and operational variation.

***3.2.2.7 Sensitivity analysis with respect to the AC system strength.*** To evaluate the performance of the proposed algorithm under variations in the AC system strength, a set of simulation scenarios was designed in which the short-circuit capacity of the AC network at the converter side was modified. In HVDC systems connected to AC networks, the strength of the AC system can significantly influence the transient behavior of line currents and voltages. Increasing the AC system strength generally reinforces the fault feeding capability and results in higher amplitudes of transient currents, whereas weakening the AC system may reduce signal magnitudes and alter the dynamic characteristics of the system response. Therefore, assessing the performance of the protection algorithm under both strengthened and weakened AC system conditions is important in terms of detection stability, classification accuracy, and fault-location precision. For this purpose, three different scenarios were investigated. In the first scenario, the AC system strength was increased by 30% relative to its nominal value in order to emulate a stronger network condition. This modification was introduced at t = 0.5 s so that the system could reach a new steady-state operating point. Subsequently, a PTG fault was applied at t = 0.75 s at a distance of 120 km from terminal T_1_, corresponding to approximately 40% of the 300 km transmission-line length. The proposed algorithm detected the fault at t = 0.7526 s, corresponding to a detection delay of 2.6 ms. The classification stage also operated correctly, and the fault was accurately identified as a PTG fault. The estimated fault distance was calculated as 121.5 km, which corresponds to a location error of approximately 0.5% of the line length. These results indicate that even under strengthened AC system conditions, the proposed algorithm is capable of detecting faults rapidly and accurately.

In the second scenario, the AC system strength was reduced by 30% relative to its nominal value in order to represent a weaker network condition. This change was applied at t = 1.2 s in the system model. After the system reached a stable operating condition, an NTG fault was applied at t = 1.5 s at a distance of 210 km from terminal T1, corresponding to 70% of the line length. The algorithm detected the fault at t = 1.5030 s, indicating a detection delay of approximately 3.0 ms. In this scenario, the classification stage also performed correctly, and the fault type was accurately identified as NTG. The estimated fault distance was 208.8 km, corresponding to a location error of approximately 0.4% of the line length. The results of this scenario demonstrate that even under weakened AC system conditions and reduced transient signal amplitudes, the proposed algorithm can still reliably detect the fault and accurately determine its location.

In the third scenario, the AC system was maintained under nominal operating conditions without any modification in network strength. In this case, a PTN fault was applied at t = 1.8 s at a distance of 240 km from terminal T_1_, corresponding to 80% of the line length. The proposed algorithm detected the fault at t = 1.8032 s, corresponding to a detection delay of 3.2 ms. The classification stage also operated correctly in this scenario, and the fault was accurately identified as a PTN fault. The estimated fault distance was calculated as 238.6 km, corresponding to a location error of approximately 0.47% of the line length.

The results obtained from these scenarios demonstrate that variations in the AC system strength do not have a significant impact on the performance of the proposed algorithm. In all investigated cases, the algorithm was able to detect the fault with very short delay, and the classification stage correctly identified the fault type without error. Furthermore, the fault-location estimation error remained below approximately 0.5% of the transmission-line length in all scenarios, indicating the high accuracy of the proposed method.

Overall, these results confirm that the proposed algorithm exhibits stable performance under variations in AC system strength. Whether the AC network is strengthened or weakened, the algorithm can rapidly detect fault occurrence, correctly classify the fault type, and estimate the fault location with high accuracy. This characteristic demonstrates the reliability and flexibility of the proposed method for practical application in HVDC systems connected to AC networks with different short-circuit capacity levels.

***3.2.2.8 Sensitivity analysis with respect to sudden wind-speed variations and generation changes.*** To evaluate the performance of the proposed algorithm under variable wind-farm operating conditions, the effect of sudden wind-speed variations and the resulting changes in the generated power of the wind farm connected to the HVDC link was investigated. Since wind-speed variations directly affect the active power injected into the DC link, they can shift the system operating point, modify the pre-fault current level, and change the transient behavior of the measured signals at terminal T_1_. Therefore, this scenario was considered one of the important practical operating conditions for assessing the robustness of the proposed algorithm. In this study, the wind speed was initially set to its base value used in the reference simulation, so that the system operated at a stable operating point with a moderate generation level. Then, at t = 0.6 s, the wind speed was increased stepwise by 12%. This sudden increase shifted the wind-farm output from a moderate level toward a range close to the rated power, thereby increasing the active current injected into the DC link. Subsequently, to impose a more severe operating variation, the wind speed was suddenly reduced by 15% at t = 0.8 s. This decrease caused a noticeable reduction in the wind-farm output power and, consequently, in the current injected into the DC link. Accordingly, the proposed algorithm was evaluated under conditions in which the system experienced a sudden increase in generation followed by a rapid generation drop within a short time interval. As a result, the pre-fault current level, as well as the operating conditions of the converter and transmission line, changed noticeably. After applying these variations, and before the system fully settled to a new steady operating condition, three fault types, namely PTG, NTG, and PTP, were applied at distances of 90 km, 190 km, and 290 km from terminal T_1_, respectively. The inception times of the first, second, and third faults were 0.5 s, 0.7 s, and 0.9 s, respectively, and the fault resistance was assumed to be 100 ohms in all three cases. This setting enabled the performance of the proposed algorithm to be assessed under input conditions affected by realistic and severe generation variations caused by wind-speed fluctuations.

The simulation results show that sudden wind-speed variations and the resulting shift in the system operating point do not have a meaningful impact on the performance of the proposed algorithm. Despite changes in the pre-fault current level and transient fluctuations caused by generation variations, the algorithm detected the PTG, NTG, and PTP faults with delays of 2.69 ms, 2.93 ms, and 3.15 ms, respectively. These results indicate that the operating speed of the proposed method is preserved under dynamic network conditions.

At the classification stage, the fault type and the involved pole were correctly identified in all three scenarios, and the algorithm was able to distinguish properly among PTG, NTG, and PTP faults. For fault location estimation, the distance estimation errors for the three cases were 0.74%, 0.88%, and 0.97% of the line length, respectively. These values show that generation changes caused by wind-speed fluctuations have only a limited effect on the final accuracy of the fault-location estimation.

Overall, this sensitivity analysis demonstrates that the proposed algorithm has suitable robustness against sudden wind-speed variations and changes in wind-farm generation. It can perform fault detection, classification, and location estimation with acceptable speed and accuracy even under variable and dynamic operating conditions.

***3.2.2.9 Sensitivity analysis with respect to HVDC network size.*** To evaluate the generalization capability of the proposed protection algorithm in larger-scale networks, the performance of the method was investigated in a multi-terminal HVDC network. For this purpose, a four-bus MMC-HVDC network was considered based on the structure reported in reference [26]. In this network, four converter stations are interconnected through HVDC transmission lines, where the first converter operates in voltage-control mode and the remaining converters operate in power-control mode. To make the simulation conditions closer to practical networks, it was also assumed that one of the buses is connected to a wind farm, whose generated power is transferred to other parts of the system through the HVDC network. Increasing the number of buses and transmission paths in such a network can make transient-wave propagation more complex, increase the number of wave-reflection paths, and modify the pattern of fault currents. Therefore, evaluating the proposed method in this larger structure is important in terms of detection accuracy, classification reliability, and fault-location performance. In this study, the transmission line between Bus 1 and Bus 4 was selected as the protected line, and three fault scenarios were defined at different locations along this line. The objective of these scenarios was to assess the algorithm performance under different fault locations and fault types in a multi-terminal network. In the first scenario, the transmission-line length was set to 200 km, and a PTG fault was applied at t = 0.75 s at a distance of 60 km from Bus 1, corresponding to 30% of the line length. The proposed algorithm detected the fault at t = 0.7527 s, indicating a detection delay of 2.7 ms. In this case, the fault classification stage also operated correctly, and the fault was accurately identified as a PTG fault. The estimated fault distance was calculated as 61.2 km, which corresponds to a location error of approximately 0.6% of the line length compared with the actual fault location. In the second scenario, to examine the performance of the algorithm in a larger network condition, the transmission-line length was set to 400 km. In this case, an NTG fault was applied at t = 1.5 s at a distance of 240 km from Bus 1, corresponding to 60% of the line length. The proposed algorithm detected the fault at t = 1.5031 s, corresponding to a detection delay of 3.1 ms. In this scenario, the classification stage again operated without error, and the fault type was correctly identified as NTG. The estimated fault distance was 238.9 km, corresponding to a location error of approximately 0.55% of the line length.

In the third scenario, to evaluate the algorithm performance for a fault close to the remote end of the line, a PTN fault was applied at t = 1.8 s at a distance of 320 km from Bus 1, corresponding to 80% of the 400 km line length. The proposed algorithm detected the fault at t = 1.8033 s, which is equivalent to a detection delay of 3.3 ms. The classification stage also performed correctly in this case, and the fault was identified as PTN. The estimated fault distance was calculated as 318.2 km, corresponding to a location error of approximately 0.45% of the line length.

A comparison of the results obtained from these scenarios shows that the proposed algorithm maintains stable performance in a four-bus HVDC network. The fault detection delay remained below approximately 3.5 ms in all scenarios, indicating the high speed of the proposed method in fault detection. In addition, fault classification was performed correctly for all PTG, NTG, and PTN faults, and no misclassification was observed. In terms of fault location, the estimation error remained below approximately 0.6% of the line length in all scenarios, demonstrating the suitable accuracy of the proposed method in locating faults even in larger and multi-terminal networks. Overall, the results of this analysis indicate that increasing the HVDC network size and adopting a multi-bus structure do not have a significant adverse effect on the performance of the proposed algorithm. Even in the presence of renewable generation sources such as a wind farm and longer transmission paths, the algorithm can still detect faults rapidly, correctly classify the fault type, and estimate the fault location with acceptable accuracy. These results confirm the generalization capability and practical effectiveness of the proposed method for large-scale and multi-terminal HVDC networks.

***3.2.2.10 Sensitivity analysis with respect to converter operating and control conditions.*** To evaluate the robustness of the proposed algorithm against changes in the operating conditions of the converters, a sensitivity scenario was considered in which the operating mode of the AC/DC converter at terminal T_1_ was modified. As explained in the paper, the proposed algorithm operates only based on local measurements at terminal T_1_, including the positive- and negative-pole voltages and currents. It estimates the fault location using the initial transient response of the currents and a resistive line model that includes the series resistance of each pole as well as the mutual resistive effect between the two poles. Therefore, changing the converter control regime can be regarded as a suitable test for assessing the performance of the method under variations in the system operating point.

In the base operating condition, converter T_1_ is responsible for regulating the DC-link voltage, and the reference value of the link voltage is set to its nominal value of 1.0 p.u. To introduce a change in the operating condition, the structure of the converter outer control loop was modified at t = 0.20 s, and the responsibility for regulating the DC-link voltage was transferred from terminal T_1_ to the opposite terminal. Accordingly, converter T_1_ was changed from DC-voltage control mode to active-power control mode, controlling the active power injected into the DC link.

To create different operating points, the DC-link voltage reference was considered at three operating levels: 0.9 p.u., 0.8 p.u., and 1.1 p.u., corresponding to a 10% decrease, a 20% decrease, and a 10% increase in the DC-link voltage relative to the nominal value, respectively. To evaluate the performance of the algorithm under these conditions, as in the other sensitivity-analysis scenarios, three fault types, namely PTG, NTG, and PTP, were applied at distances of 90, 190, and 290 km from terminal T_1_, respectively. The inception time of all three faults was set to t = 1.0 s, and the fault resistance was assumed to be 100 ohms in all cases in order to simulate medium-resistance faults on the HVDC line. The simulation results show that, despite the change in converter operating mode and DC-link voltage level, the proposed algorithm remains capable of detecting faults within a very short time. In this scenario, the detection delays for the PTG, NTG, and PTP faults were 2.61, 2.84, and 3.07 ms, respectively. These values indicate that the fast-operating speed of the algorithm is preserved even when the converter operating point changes.

At the classification stage, the fault type and the involved pole were correctly identified in all three cases, and the algorithm was able to distinguish properly among PTG, NTG, and PTP faults. In terms of fault location, the results show that the distance estimation accuracy remained within an acceptable range. The fault-location errors for the three cases were 0.68%, 0.79%, and 0.91% of the line length, respectively.

These results indicate that changing the converter control mode and the DC-link voltage level has only a very limited effect on the performance of the proposed algorithm. The proposed method can still perform fault detection, classification, and location estimation with suitable speed and accuracy even under changes in the converter operating regime.

***3.2.2.11 Sensitivity analysis with respect to DC-link voltage deviation.*** To assess the stability and reliability of the proposed algorithm under conditions where the DC-link voltage deviates from its nominal value, the effect of DC-link voltage variations on fault detection, classification, and location estimation was evaluated. In converter-based HVDC systems, the DC-link voltage may deviate from its reference value due to changes in the generated power of sources, AC-grid disturbances, variations in converter operating conditions, or controller actions. Since the proposed algorithm uses DC-link voltage and current signals to extract transient features, variations in the DC voltage level may affect the amplitude and characteristics of the measured signals. In this scenario, the nominal DC-link voltage of the system was considered as the reference value in the simulation model, and voltage deviations were then introduced in the converter model through software-based adjustments. Specifically, three DC-link voltage conditions were investigated: a 10% increase, a 10% decrease, and the nominal condition, in order to emulate practical operating variations. In other words, if the nominal DC-link voltage is denoted by Vdc, two additional operating conditions, 1.1Vdc and 0.9Vdc, were applied in the system model. These variations were implemented by adjusting the voltage reference in the converter control loop, which consequently changed the voltage levels of the positive and negative poles of the DC link during the simulation. Under these conditions, three fault types, namely PTG, NTG, and PTN, were applied at distances of 80, 180, and 280 km from terminal T_1_, respectively. The fault inception time was set to t = 1.0 s in all cases, and the fault resistance was assumed to be 100 ohms so that the influence of the DC voltage level could be evaluated independently from other parameters. By applying these changes, the amplitudes of the DC-link voltage and current signals measured at terminal T_1_ differ from those in the nominal case, creating different conditions for transient-feature extraction by the algorithm. The simulation results show that DC-link voltage deviation has only a limited effect on the performance of the proposed algorithm, and the proposed method can still identify faults with suitable speed and accuracy. In this scenario, the fault detection delays for the investigated cases were in the range of 2.6 to 5.9 ms, specifically 2.6, 4.1, and 5.9 ms. These values indicate the stability of the algorithm against variations in the system voltage level. In addition, at the fault classification stage, the fault type and the involved pole were correctly identified in all cases, and no misclassification was observed. Regarding fault location, the results show that the distance estimation error under DC-link voltage deviation remained within the range of 0.9% to 2.3% of the line length. This indicates that although variations in the DC voltage level can affect the amplitudes of the measured signals, the time-domain and frequency-domain structures of the transient components used by the algorithm are largely preserved. Therefore, the fault-location accuracy is only moderately affected. Overall, the results of this sensitivity scenario demonstrate that the proposed algorithm has suitable robustness against DC-link voltage deviations of approximately ±10% from the nominal value. Even when the system voltage level changes, the algorithm remains capable of performing fault detection, classification, and location estimation with acceptable accuracy. This feature confirms the applicability of the proposed method under practical operating conditions of HVDC systems, where DC-link voltage fluctuations are unavoidable.

The sensitivity-analysis results presented in Fig 14 evaluate the performance of the proposed protection method against a wide range of practical uncertainties, including variations in operating conditions, network parameters, and measurement errors. To provide a structured assessment, the algorithm performance is analyzed at three main levels: fault detection, fault-type classification, and fault location. First, the results show that the fault-detection stage has a very high level of stability. In all investigated scenarios, fault occurrence was identified without any missed detection or false detection, achieving a detection accuracy of 100%. This behavior indicates that the indices extracted from the transient components of current and voltage maintain a sufficient signal-to-noise ratio to activate the protection logic, even in the presence of disturbances such as CT saturation, measurement-noise injection, line-parameter variations, and changes in network operating conditions. In addition, the distribution of detection delays shows that the average detection time is approximately 3.07 ms, and in most scenarios it remains below 3.5 ms. This indicates that network-parameter variations mainly cause only limited attenuation of the transient amplitudes and do not fundamentally distort the temporal structure of the signals. Second, the fault-classification stage, similar to the detection stage, demonstrates considerable stability. In all investigated scenarios, including high-resistance faults, variations in AC system strength, changes in converter control modes, and modifications in the HVDC network structure, the algorithm successfully distinguished the fault type and the involved pole. This result shows that the indices used for classification have limited dependence on the system operating point and rely mainly on the relative pattern of voltage–current responses in the two poles. Consequently, operating-condition variations or measurement uncertainties do not make the decision-making pattern of the algorithm ambiguous. Third, the fault-location performance is examined. This stage is naturally more sensitive to network variations than the two previous stages, because fault-distance estimation is directly dependent on the propagation and attenuation characteristics of fault waves along the DC line. The results show that the average fault-location error is approximately 0.75% of the line length, and in most scenarios it remains below 1%, confirming the high accuracy of the proposed method in estimating the fault position. However, the largest increase in location error is observed in the DC-link voltage deviation scenario, where the fault-location error increases to approximately 2.3%. This behavior can be attributed to changes in the energy level of the initial transients and variations in the voltage–current ratio at the instant of fault inception, which directly affect the parameters used in the fault-location algorithm. In contrast, scenarios such as changes in AC system strength, line-voltage level, and transmission-line parameters have only a limited effect on location accuracy and show the smallest deviation from the nominal performance of the algorithm. Overall, the results presented in Fig 14 demonstrate that the proposed method is structurally robust against a wide range of model uncertainties and operating conditions. Maintaining full accuracy in the detection and classification stages, together with very limited fault-location error, indicates that the decision logic of the algorithm is mainly based on the fundamental characteristics of fault transients rather than on settings that are highly sensitive to specific network conditions. The only scenario that causes the largest performance deviation is a significant change in the DC-link voltage, which directly affects the amplitude and energy of the transients. Nevertheless, even under this condition, the algorithm performance remains within an acceptable range for HVDC protection applications.

## 4. Discussion and comparison of the proposed method with previous works

In this section, a systematic and high-fidelity comparative analysis is performed to benchmark the proposed protection framework against state-of-the-art methods in HVDC transmission line protection. The primary objective is to evaluate the technical viability and operational efficiency of different strategies under a unified evaluation framework. To this end, the discussion is structured into two main subsections: Section 4.1 provides a critical review of prominent existing methods, while Section 4.2 focuses on a detailed analytical evaluation of the proposed strategy.

The core of this comparison is centered on Table 2, which provides a multi-dimensional assessment based on fourteen distinct evaluation criteria. These criteria are strategically divided into two categories:

**Table 2:** Comprehensive analytical framework for the evaluation and comparison of HVDC protection methods.

References	Qualitative and quantitative evaluation indicators													
	Index 1	Index 2	Index 3	Index 4	Index 5	Index 6	Index 7	Index 8	Index 9	Index 10	Index 11	Index 12	Index 13	Index 14
**[3]**	Point-to-point VSC‑HVDC transmission line	Time-domain numerical method	Fault location	Location criterion using VIM solution, voltage waveform similarity and bisection refinement	Voltage and current	Based on measurements from two buses	Fault location, fault type and transition resistance	Need for both-end data and accurate line parameters	Medium to high	No faults detected	No faults detected	No fault classification	0.66%	0.95%
**[4]**	Current‑source‑ converter‑based HVDC	Machine learning	Fault detection + fault classification	Use of machine‑learning algorithms for fault pattern detection and classification	Voltage and current	single bus	Fault resistance and fault location	Dependence on training‑data quality	Medium	4.22ms	6.69ms	100%	No fault location	No fault location
**[6]**	Meshed DC grid	Signal processing + machine learning	Fault detection + fault classification + fault location	Feature extraction using wavelet transform and classification using an optimized neural network	Voltage and current	single bus	Fault resistance and operating conditions	Sensitivity to quality of feature extraction	Medium	3.5ms	5.36ms	98%	0.88%	1.22%
**[9]**	HVDC transmission line	Deep learning	Fault detection	FFT‑based signal transformation and detection using CNN	Current	single bus	Noise and fault resistance	Requirement for extensive training data	High	2.02ms	4.6ms	No fault classification	No fault location	No fault location
**[11]**	VSC‑based multi‑terminal HVDC	Deep learning + machine learning	Fault detection + fault classification + fault location	Feature extraction using HHT and hybrid CNN–SVM classification	Current	single bus	Fault resistance and fault location	High computational complexity of feature extraction	Medium to high	3.66ms	4.02ms	100%	0.86%	1.23%
**[14]**	VSC‑based HVDC	Traveling waves	Fault detection + fault location	Traveling‑wave‑based fault location using Harsdorf distance	Voltage and current	single bus	High‑resistance faults	Need for high sampling rate	Medium	1.89ms	2.89ms	No fault classification	0.33%	0.88%
**[16]**	Bipolar HVDC	Traveling waves	Fault detection + fault classification + fault location	Traveling‑wave analysis using redundant discrete wavelet transform	Voltage	single bus	Fault distance and signal conditions	Sensitivity to signal noise	Medium	4.11ms	5.66ms	94%	0.89%	1.02%
**[17]**	VSC‑based HVDC	Fault loop analysis / electrical quantity analysis	Fault point label	Fault loop analysis considering protection-action-induced voltage/current characteristics; integral-feature-based criterion	Voltage and current	Based on local measurements from a single bus	Fault resistance, fault type, and operating conditions	Limited to converter-area faults and simulation-based validation	Medium	No faults detected	No faults detected	No fault classification	NA	NA
**Proposed Method**	Bipolar HVDC connected to a wind farm	Signal processing	Fault detection + fault classification + fault location	Extraction of voltage and current transient features for local DC line protection without communication	Voltage and current	Based on local measurements from a single bus	Fault resistance, fault distance, and system operating conditions	Validation limited to simulation environment	Medium	3.32ms	4.96ms	100%	0.69%	0.98%

- Qualitative Indicators (Criteria 1–9): These address the structural and conceptual attributes of the protection schemes, such as the modeling domain, dependency on communication links, and sensitivity to topological constraints.
- Quantitative Indicators (Criteria 10–14): These provide a rigorous numerical evaluation of performance, focusing on statistical measures such as mean and maximum detection delays, classification fidelity, and the dispersion of fault location errors.

By synthesizing these indicators, this section highlights the fundamental gap in existing literature—namely, the trade-off between computational simplicity and detection robustness. While many previous works excel in isolated stages (e.g., only detection or only location) or rely on idealized assumptions such as synchronized dual-end measurements, the proposed method is evaluated for its ability to provide a unified, communication-independent solution. Ultimately, this comparative framework demonstrates that the contribution of this study lies in achieving a robust, low-complexity protection architecture that maintains high precision under realistic non-ideal operating conditions, bridging the gap between theoretical modeling and practical grid implementation.

### 4.1 Analysis of previous reliable references

To systematically analyze previous research related to HVDC system protection, Table 2 has been prepared to provide a comprehensive analytical comparison of the protection approaches proposed in different studies. The table summarizes the key characteristics of each method and highlights the major similarities and differences among them. In this comparison framework, fourteen evaluation criteria are considered. The first nine criteria are qualitative indicators that describe the conceptual structure, analytical framework, and practical characteristics of the protection methods. The remaining five criteria are quantitative indicators that evaluate the numerical performance of the protection algorithms based on simulation results. The quantitative indicators are calculated using the results of one hundred symmetrical fault simulation scenarios. In these simulations, faults are applied at different locations along the transmission line, at different time instants, and with different fault resistances and fault classes. The performance of the protection algorithm is evaluated for each scenario, and the corresponding statistical measures are extracted from the obtained results. The evaluation criteria used in this comparison are described as follows:

**1. First Criterion – Type of System Topology**

This criterion specifies the structure of the HVDC network in which the protection method has been developed or tested. The topology may include bipolar HVDC systems, multi-terminal HVDC configurations, or meshed DC grids. Since the transient behavior of faults differs significantly across these structures, the system topology plays a critical role in determining the design and performance of the protection method.

**2. Second Criterion – Solution Domain**

This criterion identifies the analytical domain or methodological framework adopted by the protection approach. Typical domains include traveling-wave-based methods, signal-processing-based approaches, machine learning or deep learning techniques, and model-based or impedance-based analytical methods. The selected domain directly influences the computational complexity, response speed, and reliability of the protection scheme.

**3. Third Criterion – Research Objective and Protection Stages**

This criterion describes the main protection functions addressed by the study. These functions generally include three essential stages: fault detection, fault classification, and fault location. While some studies focus on a single protection stage, others attempt to integrate multiple stages within a unified framework.

**4. Fourth Criterion – Innovation and Scientific Contribution**

This criterion highlights the primary scientific contribution or innovative aspect of the research. Innovations may include the development of new protection algorithms, extraction of novel signal features, integration of multiple analytical techniques, or improvements in detection accuracy and operational speed.

**5. Fifth Criterion – Required Implementation Data**

This criterion identifies the type of measurement signals required for implementing the protection algorithm. These signals may include voltage measurements, current measurements, or a combination of both. Methods requiring fewer or simpler measurements are generally more practical and cost-effective for real-world deployment.

**6. Sixth Criterion – Communication Link Requirement**

This criterion indicates whether the protection method requires communication between the terminals of the transmission line or whether it can operate using only local measurements. Protection methods that do not depend on communication links are typically considered more reliable and easier to implement in practical systems.

**7. Seventh Criterion – Sensitivity Evaluation**

This criterion assesses the robustness of the protection method under variations in system parameters and operating conditions. These variations may include fault resistance changes, different fault locations, measurement noise, operating condition variations, and the integration of renewable energy sources. Higher robustness indicates greater practical reliability.

**8. Eighth Criterion – Main Limitations of the Study**

This criterion describes the main limitations, assumptions, or restrictions associated with the proposed method in each study. For example, a method may only be valid for specific fault types, may assume ideal measurement conditions, or may exhibit reduced accuracy under high-noise environments.

**9. Ninth Criterion – Operational and Computational Complexity**

This criterion evaluates the implementation difficulty of the protection algorithm. The complexity is determined based on factors such as the number of processing stages, computational burden, use of advanced filters or heavy algorithms, and the required decision-making time. The complexity level is typically categorized as low, medium, or high.

**10. Tenth Criterion – Average Fault Detection Delay**

This quantitative indicator represents the average time delay of the protection algorithm in detecting the occurrence of a fault. The value is obtained by evaluating the algorithm performance across one hundred different symmetrical fault simulation scenarios. These scenarios include faults occurring at different locations along the transmission line, at different time instants, and with different fault resistances and fault classes. The detection delay obtained in each scenario is recorded and the average value is calculated. The unit of this index is milliseconds (ms).

**11. Eleventh Criterion – Maximum Fault Detection Delay**

This criterion represents the maximum detection delay observed among the same one hundred simulated fault scenarios used in Criterion 10. While Criterion 10 evaluates the average response time of the algorithm, this index captures the worst-case detection delay, providing an indication of the upper bound of the protection response time.

**12. Twelfth Criterion – Fault Classification Accuracy**

This index evaluates the percentage accuracy of the protection algorithm in correctly identifying the fault class. Using the same one hundred symmetrical fault simulation scenarios, the algorithm determines whether the fault occurs on the positive pole or the negative pole of the HVDC line. The classification accuracy is calculated as the percentage of correctly identified fault classes among all scenarios.

**13. Thirteenth Criterion – Average Fault Location Estimation Error**

This criterion represents the average error in estimating the fault location with respect to a reference terminal of the HVDC transmission line. For each of the one hundred simulation scenarios, the difference between the estimated fault location and the actual fault location is calculated. The average value of these errors is then computed to represent the overall estimation accuracy of the proposed method.

**14. Fourteenth Criterion – Maximum Fault Location Estimation Error**

This index represents the maximum location estimation error observed among the one hundred simulated scenarios. Similar to Criterion 11, which evaluates the worst-case detection delay, this indicator reflects the worst-case performance of the algorithm in estimating the fault location along the transmission line.

Overall, Table 2 provides a comprehensive analytical framework for evaluating and comparing different HVDC protection methods. By considering both qualitative and quantitative criteria, it becomes possible to identify the strengths, limitations, and practical applicability of existing approaches and to clearly demonstrate the improvements achieved by the proposed protection method.

### 4.2 Analytical evaluation of the proposed protection method

A detailed examination of the results summarized in Table 2 indicates that the superiority of the proposed method is not limited to marginal numerical improvements in accuracy, but rather stems from its underlying protection architecture. While several recent approaches based on machine learning and deep neural networks, such as [4,9], and [11], report classification or detection accuracies approaching 100%, their decision-making process fundamentally relies on statistical pattern recognition within the space of training data. In contrast, the proposed method adopts a physics-informed framework built upon a distributed parameter model of the DC line. This distinction becomes particularly significant under practical operating uncertainties, including variations in fault resistance, measurement noise, parameter deviations, and changing operating points. Data-driven models may exhibit sensitivity to distribution shifts or unseen operating scenarios, whereas the proposed formulation preserves its stability by explicitly embedding the physical behavior of fault propagation into the decision process.

From a quantitative perspective, the statistical indicators reported in Table 2 further confirm the robustness of the proposed scheme. In comparison with differential-equation-based time-domain approaches [3] and frequency-spectrum-based techniques [5], which often involve considerable computational burden and sensitivity to signal preprocessing stages, the proposed method achieves a lower average fault location error together with a significantly reduced maximum estimation error. Importantly, the reduction in maximum error and dispersion is not merely a statistical refinement; in practical HVDC protection, worst-case performance is often more critical than average accuracy. A protection algorithm that performs well on average but exhibits large deviations under certain scenarios cannot be considered reliable for field deployment. The confined spread of location error obtained by the proposed scheme therefore reflects a higher degree of operational reliability.

Regarding response speed, which is a decisive factor in limiting DC fault transients, the proposed algorithm demonstrates a short detection time in the millisecond range, satisfying the stringent requirements of VSC-HVDC systems. Unlike traveling-wave-based methods [14]–[16], which depend on high-frequency signal components, precise time synchronization, and often communication-assisted measurements, the proposed strategy operates exclusively on local measurements without requiring external communication links. This structural independence eliminates vulnerabilities associated with communication latency and synchronization errors, thereby enhancing dependability under realistic grid conditions. Moreover, integrating fault detection, classification, and location within a unified analytical framework reduces computational redundancy compared to multi-stage or hybrid schemes, facilitating real-time implementation in practical protective relays.

Overall, the collective qualitative and quantitative evidence presented in Table 2 demonstrates that the proposed method achieves a balanced trade-off among response speed, location accuracy, computational feasibility, and structural robustness. This combination of attributes positions it as a comprehensive and practically viable solution for modern multi-terminal HVDC networks, where fast, accurate, and reliable fault management is essential.

## 5. Final summary and conclusions

Section 5 provides a comprehensive synthesis of the research findings and the proposed analytical protection framework for wind-farm-integrated HVDC lines. First, a high-level summary of the study is presented, highlighting the core achievements in analytical modeling and the development of the multi-stage detection, classification, and location algorithms. This is followed by a detailed conclusion that evaluates the framework’s performance using key numerical metrics derived from extensive simulations, including fault location accuracy, response time, and robustness under adverse conditions such as CT saturation and measurement noise. Subsequently, the practical implementation requirements and engineering significance of the method are analyzed, with a particular focus on its communication-independent architecture, low computational complexity, and the systematic threshold determination procedure. Finally, the chapter outlines strategic research directions for further evolving this methodology within the context of next-generation, multi-terminal HVDC grids.

### 5.1 Summary

This paper addresses the critical challenge of achieving fast and reliable protection for HVDC transmission lines, particularly in the context of integrating renewable energy sources like wind farms. Recognizing that prevailing protection schemes often depend on either dual-ended measurement with dedicated communication links or complex data-driven algorithms requiring extensive training and sophisticated signal processing, this work first provides a comprehensive review of significant existing fault detection and location methodologies for HVDC lines. The inherent limitations of these approaches—including communication delays, computational burden, sensitivity to dynamic operational conditions, and reliance on large datasets—were thoroughly analyzed. This review underscores a persistent need for a protection solution that is inherently simple, rapid, communication-independent, and grounded in the fundamental physical principles of the HVDC system. In response to these identified gaps, this study proposes a novel three-stage analytical framework for HVDC line protection, encompassing fault detection, fault classification, and precise fault location. Our approach is founded upon a robust modeling of the HVDC transmission line’s transient behavior using the distributed parameter line model. This foundational model enables the derivation of precise analytical relationships governing wave propagation and current dynamics during fault events. Leveraging these derived relationships, several key indices have been defined based on current components measured solely at the local terminal. These indices are specifically engineered to accurately detect the fault inception instant and reliably classify fault types across diverse operating scenarios. Building upon this model, a communication-independent algorithm has been developed to estimate fault location distance from the measurement terminal. To rigorously validate the performance and robustness of the proposed method, a detailed simulation model of an HVDC transmission system integrated with a wind farm was meticulously developed in MATLAB/Simulink. This model incorporates a wide array of fault scenarios, including pole-to-ground and pole-to-pole faults at various distances along the line, under diverse operating conditions. The simulation analysis systematically evaluated the impact of critical parameters such as fault location, fault resistance, system operational states, and fluctuating wind power generation. Furthermore, to ascertain the algorithm’s efficacy and its standing in the field, a comprehensive sensitivity analysis and a detailed performance comparison with several state-of-the-art approaches from the literature were conducted. These evaluations focused on critical metrics including accuracy, computational complexity, and communication independence. The results compellingly demonstrate that the proposed method, despite its structural simplicity and sole reliance on local measurements, achieves competitive and often superior performance in fault detection and location compared to existing reference methods. In summary, this research presents a coherent and practical framework for HVDC line protection by integrating rigorous analytical modeling, a novel algorithm based on local measurements, and thorough validation through extensive simulations and comparative analyses. This work aims to overcome the limitations of conventional methods and contribute significantly to the advancement of more reliable and efficient protection solutions for future DC power networks.

### 5.2 Conclusion

The analytical framework developed in this study provides a robust solution to the protection challenges of HVDC transmission lines integrated with wind farms. By leveraging the distributed-parameter model, we derived precise analytical relationships between the transient current response at the local terminal and the spatial fault location. The efficacy of the proposed method was rigorously validated through a detailed simulation model, revealing several key technical outcomes:

- Fault Detection and Classification Performance: The indices derived from the transient current response demonstrated high sensitivity and speed. Simulation results confirm that the proposed algorithm consistently detects fault inception within 2.86 to 3.21 ms, regardless of the fault type. Furthermore, the framework accurately identifies fault classification and the involved pole in 100% of the examined scenarios.
- Fault Location Accuracy: The analytical fault-location algorithm achieved high-precision estimation without the need for two-terminal synchronization. The simulation results indicate fault location errors of merely 0.68%, 0.76%, and 0.92% for various scenarios at 15 km, 150 km, and 285 km, respectively. This confirms a superior level of precision that remains relatively uniform across the entire line length.
- Robustness under Practical Conditions: Sensitivity analysis validated the method’s resilience against critical operational variables. Even under severe CT saturation—where magnetizing inductance drops to 20–30% of its linear value—the proposed algorithm effectively mitigates distortions and maintains its protective functions. Moreover, under measurement uncertainty with a 30 dB SNR (representing a 3.16% effective noise level), the algorithm successfully preserved its functional stability, proving its viability for real-world implementation.

Summary of Technical Contributions:

- Analytical Foundation: Establishing a mathematical link between local transient current measurements and the physical fault location based on distributed-parameter dynamics.
- Communication Independence: Successfully eliminating the dependence on two-terminal data exchange and precise time synchronization, thereby addressing one of the most critical vulnerabilities in modern HVDC protection.
- Computational Efficiency: Proposing an algorithmic structure with low computational burden, facilitating seamless integration into existing digital protective relay platforms.
- Superior Robustness: Demonstrating reliable fault detection and location even under challenging scenarios such as high fault resistances, varying wind power penetrations, and significant CT saturation-induced harmonic distortions.

In conclusion, this research presents a coherent, fast, and reliable protection framework that bridges the gap between theoretical physical modeling and practical relay implementation. As DC networks continue to expand and renewable energy integration increases, this local-measurement-based approach offers a scalable, robust, and cost-effective solution for ensuring the stability and security of future HVDC power transmission systems.

### 5.3 Practical implementation requirements and limitations of the proposed scheme

In this section, the practical implementation requirements and limitations of the proposed scheme are discussed. One of the most prominent features of this research is that the algorithm relies exclusively on local-terminal measurements, thereby eliminating the need for remote-end data or high-speed communication infrastructure. Unlike conventional protection schemes that depend on two-terminal data exchange, dedicated communication links, or precise time synchronization, the proposed method operates independently of communication networks. This characteristic significantly reduces implementation complexity and inherently mitigates the system’s vulnerability to communication latency, signal noise, and link failures, which are primary bottlenecks in modern HVDC line protection. Regarding the instrumentation requirements, a sampling frequency of 2 kHz for voltage and current transducers is employed in this framework, which is well within the capabilities of contemporary Intelligent Electronic Devices (IEDs) used in high-voltage substations. From a computational perspective, unlike AI-driven methods or advanced signal processing techniques (e.g., wavelet transforms or empirical mode decomposition) that require intensive matrix operations and high memory overhead, the analytical formulation derived in this paper is based on fundamental algebraic operations. This ensures that the algorithm can be executed in real-time on standard microprocessors or Field-Programmable Gate Arrays (FPGAs) with minimal computational burden. Concerning the threshold parameters, it should be noted that the performance of the proposed scheme is inherently dependent on the defined thresholds. This paper presents a systematic, physics-based procedure for calculating these four critical thresholds. Due to the per-unit (pu) normalization, the extracted thresholds possess inherent scalability and can be applied to similar network configurations. However, it must be emphasized that the algorithm is strictly tied to these calculated thresholds; thus, any manual alteration of these values without following the systematic procedure outlined in this study would degrade the algorithm’s performance. Finally, the applicability of this scheme is specifically focused on the detection, classification, and location of short-circuit faults (pole-to-ground and pole-to-pole). It is not designed to address other disturbances, such as open-phase faults. This clarity in defining the scope and limitations, while highlighting the advantages of communication independence and computational efficiency, establishes a robust and technically grounded framework for the practical application of the proposed method.

### 5.4 Future research directions

Considering the framework presented in this research, several research directions can be defined as future topics with emphasis on software-level development of the algorithm and with consideration of wind-farm presence in HVDC networks:

- Optimization of protection thresholds by taking into account variations in wind-farm power output
- Development of the fault detection, classification, and location method for multi-terminal HVDC networks connected to multiple wind farms
- Improvement of the performance accuracy of protection algorithms in HVDC transmission lines under uncertainties caused by wind generation and considering severe wind-farm transients
- Integration of analytical indices with machine-learning methods for fault-pattern recognition

These topics can contribute to the development of protection methods compatible with HVDC networks connected to wind energy sources and provide a suitable basis for further studies.

## Abbreviations

BPNN: Back Propagation Neural Network
CSC: Current Source Converter
CT: Current Transformer
DFIG: Doubly-fed Induction Generator
DOST: Discrete Orthonormal Stockwell Transform
FDST: Fast Discrete Stockwell Transform
FDR: Fisher Discriminant Ratio
FFT: Fast Fourier Transform
HVDC: High-Voltage Direct Current
IQR: Interquartile Range
KDE: Kernel Density Estimation
KNN: K-Nearest Neighbors
LSSVM: Least Squares Support Vector Machine
MMC: Modular Multilevel Converter
MT: Multi-Terminal
NPC: Neutral Point Clamped
OED: Optimal Experimental Design
PNN: Probabilistic Neural Network
PTG: Positive Pole-to-Ground
PTN: Positive Pole-to-Negative Pole
SVM: Support Vector Machine
VSC: Voltage Source Converter
WOA: Whale Optimization Algorithm.
